# Codon and amino acid content are associated with mRNA stability in mammalian cells

**DOI:** 10.1371/journal.pone.0228730

**Published:** 2020-02-13

**Authors:** Megan E. Forrest, Otis Pinkard, Sophie Martin, Thomas J. Sweet, Gavin Hanson, Jeff Coller

**Affiliations:** 1 Center for RNA Science and Therapeutics, Case Western Reserve University, Cleveland, Ohio, United States of America; 2 Department of Genetics and Genome Sciences, Case Western Reserve University, Cleveland, Ohio, United States of America; Korea University, REPUBLIC OF KOREA

## Abstract

Messenger RNA (mRNA) degradation plays a critical role in regulating transcript levels in the cell and is a major control point for modulating gene expression. In yeast and other model organisms, codon identity is a powerful determinant of transcript stability, contributing broadly to impact half-lives. General principles governing mRNA stability are poorly understood in mammalian systems. Importantly, however, the degradation machinery is highly conserved, thus it seems logical that mammalian transcript half-lives would also be strongly influenced by coding determinants. Herein we characterize the contribution of coding sequence towards mRNA decay in human and Chinese Hamster Ovary cells. In agreement with previous studies, we observed that synonymous codon usage impacts mRNA stability in mammalian cells. Surprisingly, however, we also observe that the amino acid content of a gene is an additional determinant correlating with transcript stability. The impact of codon and amino acid identity on mRNA decay appears to be associated with underlying tRNA and intracellular amino acid concentrations. Accordingly, genes of similar physiological function appear to coordinate their mRNA stabilities in part through codon and amino acid content. Together, these results raise the possibility that intracellular tRNA and amino acid levels interplay to mediate coupling between translational elongation and mRNA degradation rate in mammals.

## Introduction

Messenger RNA (mRNA) stability is a highly regulated process, ultimately determining mRNA levels within the expressed transcriptome. A major mechanism for normal cytoplasmic mRNA degradation is initiated by deadenylation of the 3’-polyA tail. This event involves the concerted efforts of the PAN2/PAN3 complex followed by bulk deadenylation by the CCR4/CAF1 complex [1–3]. The removal of the polyA tail then triggers cleavage of the 5’-7-methylguanosine cap by the DCP1/2 decapping complex, exposing a free 5’-monophosphate group that is recognized by the highly processive 5’-3’ exonuclease XRN1 [2,4,5]. This pathway is most well defined in *Saccharomyces cerevisiae*, but all of the major factors are conserved from yeast to humans [6,7]. While deadenylation-dependent decapping is the major mechanism for decay in yeast, in mammalian systems, deadenylation-dependent 3’-5’ decay by the cytoplasmic exosome and a novel ribosome-phased endonucleolytic decay pathway also occur extensively [8–10]. Together, these data suggest that the dominant mechanisms of mRNA turnover in mammalian cells may be incompletely defined.

In recent years, multiple factors related to regulation of mRNA stability have been characterized in an effort to explain the wide variation observed in mammalian mRNA half-lives *in vivo* [11–13]. A key element in mRNA stability is ribonuclear protein (RNP) composition—for example, binding by polyA binding protein (PABP) at the 3’-end and the cap-binding protein eIF4E at the 5’-end are necessary for cytoplasmic mRNA stability [14–18]. In particular, multiple other RNA-binding proteins (RBPs) have been found to bind within the 5’- or 3’-untranslated regions (UTRs) to dynamically regulate mRNA stability under various cellular conditions, notably at instability-promoting sites such as AU-rich elements [6,10,19,20]. 3’-UTRs also commonly harbor binding sites for microRNAs, which typically accelerate mRNA decay by recruiting decay machinery and stripping stabilizing mRNP components [21–25]. While 3’-UTR-based factors are thought to represent a major regulatory mechanism in mammalian mRNA stability, variation in 3’-UTRs is not always sufficient to explain the vast repertoire of observed half-lives [26].

For decades it has been appreciated that mRNA stability and translation are highly interconnected. Broadly speaking, mRNAs that translate well are more stable than those that translate to a lesser extent. Although this phenomenon was first described in the 1970s, the underlying molecular principles that govern this intimate relationship have been a mystery until recently [27]. We previously described codon optimality within the open reading frame as a key determinant of mRNA stability in *S*. *cerevisiae*, where the proportion of “optimal” and “non-optimal” codons have large effects on overall transcript stability [28,29]. In budding yeast, the prevailing model for the distinction between “optimal” and “non-optimal” codons is thought to be driven by the balance of cognate tRNA availability versus codon demand [30]. However, additional studies have proposed a different model, where tRNA levels and codon usage within the transcriptome are largely balanced via adaptive changes in tRNA and mRNA expression; here, differences in translation efficiency between different codons are explained by imbalances between tRNA levels and amino acid usage [31]. In support of the former model, we have directly demonstrated differences in translation and ribosome occupancy over optimal versus non-optimal codons [29,32]. Therefore, codon optimality represents a critical link between translation elongation and mRNA stability, where slow translation of non-optimal mRNAs is sensed by the 5’-3’ decay pathway machinery to trigger rapid deadenylation and decay [27–29,32–35]. Similar relationships between synonymous codon content and mRNA half-life have since been demonstrated for multiple other organisms, including *Schizosaccharomyces pombe* [36], *Trypanosoma brucei* [37], *Drosophila* [38], zebrafish [39,40], and, most recently, in humans [41,42]. While the effects of ORF composition on mRNA stability appear to be broadly conserved, they appear to vary widely in magnitude among different organisms, suggesting possible differences in mechanism and underlying causes.

In this study, we show that altering optimal codon content in naturally-occurring human mRNA sequences affects their stability. Further, we investigated global relationships between codon content and mRNA stability in two separate mammalian cell lines (HeLa and Chinese hamster ovary (*Cricetulus griseus*)) and show that different codons correlate with varying effects on mRNA stability, complementing the results of recent studies [41–43]. In stark contrast to our findings in budding yeast, however, we determined that a gene’s encoded amino acid content significantly correlates with observed differences in mRNA stability. We further show that changing amino acid content is sufficient to alter reporter mRNA stability without changing optimal codon content. To investigate possible factors influencing codon- and amino acid-specific stability measures, we robustly analyze tRNA levels by tRNA-Seq and determine that differences in intracellular tRNA levels are associated with codon-based effects on stability for a subset of codons, but not for codons encoding amino acids with extreme effects on stability. Conversely, free amino acid levels may contribute to observed differences in stability for codons encoding amino acids with extreme amino acid effects. In support of these findings, analysis of codon and amino acid usage in groups of related genes demonstrates that both synonymous codon and amino acid usage are associated with observed mRNA half-lives in human cells. Finally, we compile codon-specific stability data for multiple species and find that codon and amino acid-based effects on stability show distinct patterns over different species. Taken together, these data suggest that both codon content and encoded amino acid sequence contribute to regulation of mRNA stability in endogenous mammalian genes.

## Results

### Optimal codon usage within endogenous human ORFs regulates mRNA stability

To investigate the relationship between optimal codon content and mRNA stability in human cells, we first sought to assess the relationship between mRNA stability and optimal codon content in the context of individual open reading frames using a human cell-specific codon optimality metric. Previously, we used codon-specific tRNA adaptive indices (tAIs) to define optimal and non-optimal codons, which estimate relative tRNA abundance based on tRNA gene copy number [28,29,44]; importantly, these metrics were highly predictive of differences in codon-specific stability (tAI vs CSC: *R* = 0.75, *p* = 2.58 x 10^−12^; [28]). In light of recent reports demonstrating wide variation in human tRNA expression among different cell types and disease states [45–48], estimating tRNA abundance based on tRNA gene copy number poorly approximates tRNA abundance in humans. We therefore utilized tRNA levels from previously published tRNA sequencing data in HEK293T cells [49] to calculate HEK293 cell-specific tRNA adaptive indices (tAIs) for each codon (see S2 Table for tAI values). Based on these values, we defined “optimal” codons as codons with high cognate tRNA abundance (tAI > median, 0.155), whereas codons with relatively low cognate tRNA abundance (tAI < median, 0.155) were designated “non-optimal”. Notably, codons that contain a G or C nucleotide at the 3’ position have previously been associated with increased stability in human cells [43]. However, we observed that GC3 codons were not more likely to be designated as optimal based on HEK293 tAIs (*p* = 0.4462, Fisher’s exact test; S1A Fig).

**Fig 1:**
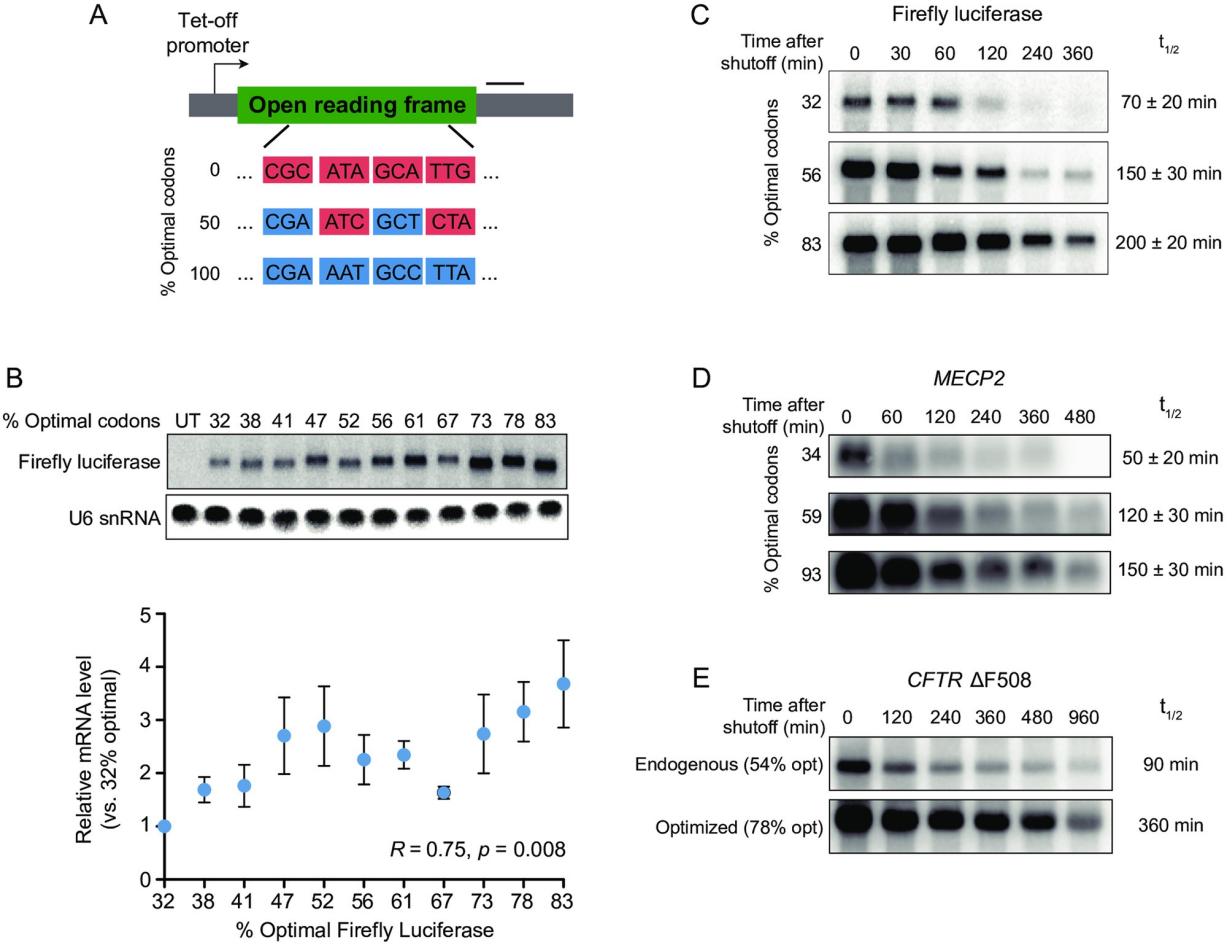
Synonymous codon content modulates mRNA stability in human cells. (A) Schematic diagram of single-gene mRNA decay reporter constructs. Tet-off promoter = minimal CMV promoter coupled to tetracycline-inducible Tet Advanced transactivator binding site for transcription shutoff. Black bar indicates Northern blot asymmetric PCR probe position. (B) (top) Northern blot analysis of steady-state Firefly luciferase mRNA levels and U6 snRNA loading control for Firefly luciferase reporters with indicated optimal codon content in HEK293 Tet-off cells. (bottom) Quantitation of northern blot signal. Error bars represent standard error of three replicates. Relative mRNA level vs. % optimal codons *R* = 0.75, *p* = 0.008 (Pearson correlation test). (C) mRNA decay analysis by northern blot for Firefly luciferase reporter constructs with indicated optimal codon content in HEK293 Tet-Off cells. Timepoints represent time elapsed after shutoff of transcription with doxycycline. t½ = half-life (min) ± standard error (n = 3). 32% optimal vs. 83% optimal difference in means = 130 min; *p* = 0.002 (two-tailed t-test). (D) mRNA decay analysis by northern blot for *MECP2* reporter constructs with indicated optimal codon content in HEK293 Tet-Off cells. Timepoints represent time elapsed after shutoff of transcription with doxycycline. t½ = half-life (min) ± standard error (n = 3). 34% optimal vs. 93% optimal difference in means = 100 min; *p* = 0.01 (two-tailed t-test). (E) mRNA decay analysis by northern blot for *CFTR* Δ5F08 endogenous sequence (53.7% optimal codons) and maximally optimized sequence (78.1% optimal codons) reporter constructs. Timepoints represent time elapsed after shutoff of transcription with doxycycline. t½ = half-life (min). See also S1 Fig for northern blot loading controls, S1 Table for northern probe sequences and reporter ORF sequences, and S2 Table for tAI and CSC values.

Using this codon optimality metric, we designed a set of 11 Firefly luciferase constructs with variable optimal codon content using a wild type Firefly luciferase input sequence (Fig 1A). Here, given that some amino acids are encoded by exclusively optimal or non-optimal synonymous codons, the 83% optimal Firefly luciferase sequence represents the theoretical maximum optimal codon composition, and the 32% optimal Firefly luciferase sequence represents the theoretical minimum optimal codon composition. Rather than randomly assembling new sequences *de novo* from a dictionary of optimal and non-optimal codons, these ORF sequences were generated by randomly “flipping” synonymous codons from optimal to non-optimal (or vice-versa) in the input sequence until the desired sequence optimality was achieved, thereby maximizing overall sequence similarity between constructs. We also noted that our variable optimality constructs generally showed increasing proportions of GC3 codon content, albeit with a narrower range (41.7–59.9%; S1B Fig). These variable optimality sequences were placed in a mammalian expression vector under the control of a Tet-off promoter consisting of a minimal CMV promoter with Tet-repressor binding sites (Fig 1A). All of the constructs were designed with identical 5’- and 3’-UTRs, allowing isolation of codon-based effects on mRNA half-life.

Analysis of steady-state Firefly luciferase mRNA abundance by agarose northern blot showed an approximately 3.5-fold increase in mRNA levels between the minimally and maximally optimized Firefly luciferase constructs, with a significant difference in overall mRNA abundance among the variable optimality reporters (*R* = 0.75, *p* = 0.008; Fig 1B). While the magnitude of change in steady-state mRNA levels more closely mirrors the change in optimal codon content, it is formally possible that increasing GC3 codon content may also be contributing to the observed changes in mRNA abundance. To directly test whether changes in mRNA levels reflect changes in stability, we shut off transcription of reporter mRNAs by doxycycline treatment in HEK293 Tet-off cells, which express a tetracycline-regulated transcription activator that is inactive in the presence of tetracycline or doxycycline [50]. Subsequent mRNA decay analysis by agarose northern blot showed an approximately 2.5-fold increase in Firefly luciferase reporter mRNA half-life when optimal codon was increased between the minimally and maximally optimized sequences (*p* = 0.002, two-tailed t-test; Fig 1C, S1C Fig), consistent with steady-state abundance measurements.

To further demonstrate the effects of optimal codon content on stability using endogenously-occurring human mRNA coding sequences, we designed an additional set of variable optimality reporters for an mRNA of similar overall length, *MECP2*. This gene is an attractive candidate for potential titration of gene expression at the level of mRNA decay, as both loss- and gain-of-function mutations in *MECP2* manifest as Rett syndrome and *MECP2* duplication syndrome, respectively [51,52]. These syndromes are characterized by overlapping severe neurological phenotypes, including intellectual disability, autism features, and seizures [53]. Analogous to our Firefly luciferase reporters, increasing optimal codon content in *MECP2* mRNA from the theoretical minimally optimized sequence (34.3% optimal codons) to the maximally optimized sequence (93.0% optimal codons) was sufficient to increase mRNA half-life by approximately 3-fold (*p* = 0.01, two-tailed t-test; Fig 1D, S1D Fig). As with our variably optimized Firefly luciferase reporters, GC3 codon content also increased by approximately 15% between the minimally and maximally optimized *MECP2* sequences (34.3% optimal = 40.5% GC3 codons; 93.0% optimal = 54.3% GC3 codons), suggesting that GC3 content could partially explain the increase in half-life; however, the magnitude of change in half-life between these reporters is more similar to the approximately three-fold increase in optimal codon content. Finally, we generated an mRNA reporter encoding human *CFTR* mRNA containing the ΔF508 mutation, the most common causative allele for cystic fibrosis [54]. Using the endogenous *CFTR* ΔF508 input sequence, we designed a maximally optimized reporter (optimized *CFTR* ΔF508 sequence = 78.1% optimal codons; endogenous *CFTR* ΔF508 = 53.7% optimal codons). Maximizing the optimal codon content of the endogenous sequence was sufficient to increase the reporter half-life by approximately 4-fold (Fig 1E, S1E Fig). Optimization was also accompanied by a large increase in GC3 codon frequency from 42.2% to 99.9%; therefore, we are unable to rule out that increasing GC3 content may also drive stabilization in this context.

Taken together, these reporter studies demonstrate that varying optimal codon content within the context of exogenously and endogenously occurring open reading frame sequences is sufficient to alter mRNA half-life in human cells, in support of recent work [41–43].

### Specific codons are globally associated with mRNA stability in mammalian cells

**Fig 2:**
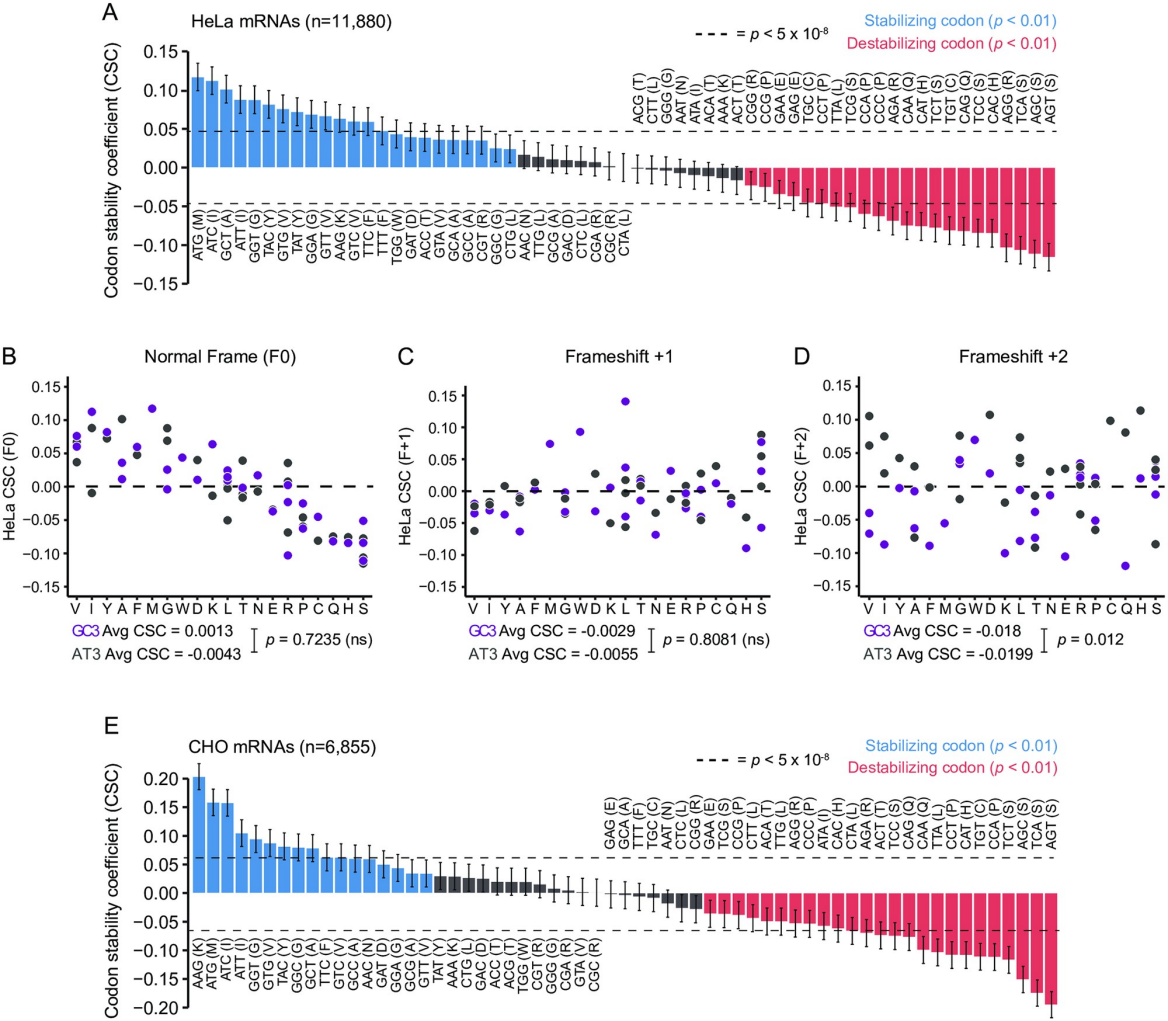
Specific codons are globally associated with mRNA stability in mammalian cells. (A) Plot of codon stability coefficients (CSC) for endogenous HeLa mRNAs (n = 11,800). Blue represents significantly stabilizing codons; red represents significantly destabilizing codons (Pearson correlation test; *p* < 0.01). Dotted lines indicate cutoff for genome-wide significance level (*p* < 5 x 10^−8^). Error bars indicate confidence interval about Pearson *R* estimate. (B-D) Plots of CSCs grouped by amino acid as calculated for (B) In-frame ORF sequences (F0), (C) ORF sequences frame-shifted by one nucleotide (F+1), and (D) ORF sequences frame-shifted by two nucleotides (F+2). Colors indicate 3’-nucleotide identity (GC3 = G or C at 3’-position, purple; AT3 = A or T at 3’-position, gray). P-values indicate results of Welch’s two-tailed t-test for difference in mean CSC between GC3 and AT3 codons. (E) Plot of codon stability coefficients (CSC) for endogenous CHO mRNAs (n = 6,855). Blue represents significantly stabilizing codons; red represents significantly destabilizing codons (Pearson correlation test; *p* < 0.01). Dotted lines indicate cutoff for genome-wide significance level (*p* < 5 x 10^−8^). Error bars indicate confidence interval about Pearson *R* estimate. See also S2 Table for CSC values and statistical data and S3 Table for half-life data and coding sequence sources.

Having established that optimal codon content affects mRNA stability in the context of individual reporter constructs, we next sought to explore the specific relationship between codon occurrence and mammalian mRNA stability transcriptome-wide. We first obtained half-life data for 11,880 wild type HeLa (human cervical adenocarcinoma) transcripts measured by 5’-bromo-uridine (BrU) immunoprecipitation chase-deep sequencing analysis (BRIC-Seq) [55,56]. We then calculated codon occurrence to mRNA stability correlation coefficients (hereafter referred to as “codon stability coefficients” or CSCs) by obtaining the Pearson correlation coefficient (*R*) between half-life and codon frequency for each of the 61 non-stop codons [28]. Based on this analysis, we observed that 23/61 codons (38%) demonstrated significant stabilizing effects at a 95% confidence level (*R* > 0, *p* < 0.01), where 13/61 (21%) reached the genome-wide significance level (*p* < 5 x 10^−8^; Fig 2A). Conversely, 21/61 codons (34%) showed significant destabilizing effects (*R* < 0, *p* < 0.01), where 16/61 (26%) demonstrated genome-wide significance (*p* < 5 x 10^−8^; Fig 2A). Taken together, these results suggest that specific codons contribute to overall transcript stability in human cells, in agreement with previous studies [41–43]. Importantly, these effects appear to be dependent on the correct reading frame, where re-calculation of CSCs on frameshifted coding sequences resulted in widespread changes in CSC measurements (Fig 2B–2D). Direct comparison of our HeLa CSC values to a parallel study [41] demonstrated significant similarities in the overall range of observed CSC values, albeit with widespread differences (*R* = 0.6, *p* = 3.0 x 10^−7^, Pearson correlation test; S1F Fig); these discrepancies may reflect differences in our half-life determination methods and analyses. Further, we noted that codons containing a G or C at the 3’-nucleotide position (GC3 codons) were not significantly more stabilizing than codons containing an A or T at the 3’-nucleotide position (AT3 codons) in our dataset, in contrast to an additional parallel study in humans (Fig 2B) [43].

Similarly, we performed global mRNA half-life analysis in Chinese Hamster Ovary (CHO) cells for 6,855 mRNAs using a 5-ethynyluridine (5-EU) metabolic labeling approach coupled to deep RNA sequencing (5-EU-Seq) and calculated codon stability coefficients as above. Similar to our global half-life analysis in HeLa cells, we observed significant enrichment of 17/61 codons (28%) in stable transcripts (*R* > 0, *p* < 0.01) and 25/61 (41%) in unstable transcripts (*R* < 0, *p* < 0.01) (Fig 2E); of these codons, 11/61 (18%) were significantly stabilizing and 18/61 (29%) were significantly destabilizing at the genome-wide significance level (*p* < 5 x 10^−8^). Indeed, 43/61 (70%) codons showed matching stabilizing or destabilizing effects between both datasets with similar magnitude (S1G Fig; *R* = 0.88, *p* < 2.2 x 10–16, Pearson correlation test). These similarities hint at possible conserved mechanisms of open reading frame-based regulation of mRNA stability among mammals.

Taken together, these data suggest that codon content within mRNA transcripts can positively or negatively impact overall transcript stability in mammalian cells, in agreement with recent studies [41–43].

### Encoding of specific amino acids contributes to mammalian mRNA stability

**Fig 3:**
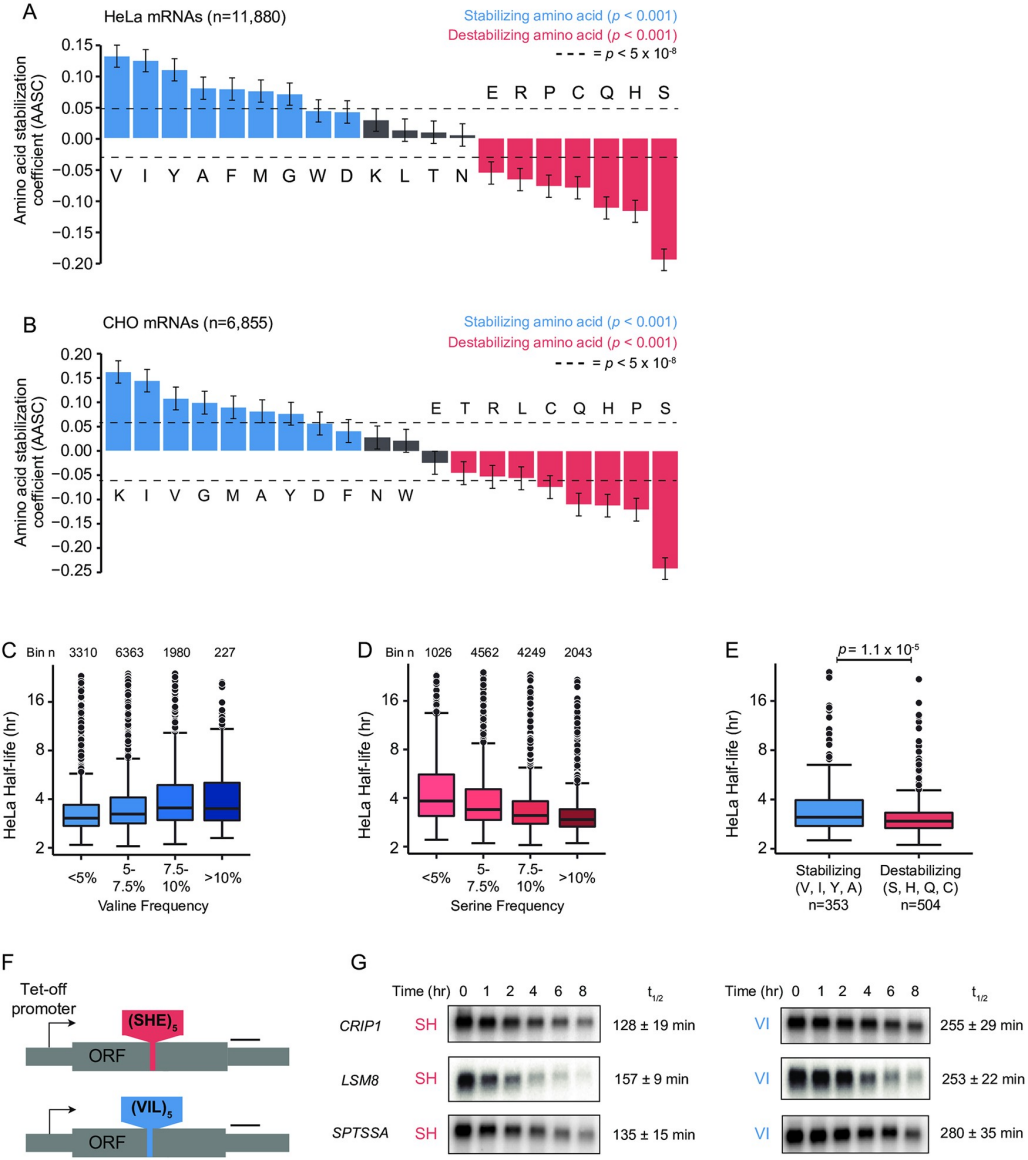
Specific amino acids are globally associated with mRNA stability in mammalian cells. (A) Plot of amino acid stabilization coefficients (AASC) for endogenous HeLa mRNAs (n = 11,800). Blue represents significantly stabilizing amino acids; red represents significantly destabilizing amino acids (Pearson correlation test; *p* < 0.001). Dotted lines indicate cutoff for genome-wide significance level (*p* < 5 x 10^−8^). Error bars indicate confidence interval about Pearson *R* estimate. (B) Plot of amino acid stabilization coefficients (AASC) for endogenous CHO mRNAs (n = 6,855). Blue represents significantly stabilizing amino acids; red represents significantly destabilizing amino acids (Pearson correlation test; *p* < 0.001). Dotted lines indicate cutoff for genome-wide significance level (*p* < 5 x 10^−8^). Error bars indicate confidence interval about Pearson *R* estimate. (C) Boxplots of HeLa mRNA half-life distributions binned by valine frequency. Number of transcripts in bin is indicated above each boxplot. Overall difference in means *p* < 2.2 x 10^−16^ (Kruskal-Wallis test). (D) Boxplots of HeLa mRNA half-life distributions binned by serine frequency. Number of transcripts in bin is indicated above each boxplot. Overall difference in means *p* < 2.2 x 10^−16^ (Kruskal-Wallis test). (E) Boxplots of HeLa mRNA half-life distributions comparing transcripts containing homopolymeric repeats of the four most highly destabilizing amino acids (S, H, Q, and C) vs. transcripts containing the four most highly stabilizing amino acids (V, I, Y, and A) as defined by AASC (Fig 3A); *p* = 1.1 x 10^−5^ (Wilcoxon test). (F) Schematic diagram of amino acid stretch insertion reporter constructs for single-gene mRNA decay analysis. Tet-off promoter = minimal CMV promoter coupled to tetracycline-responsive Tet Advanced transactivator binding site for transcription shutoff. Black bar indicates Northern blot asymmetric PCR probe position. (G) Transcription shutoff/northern mRNA decay analysis of amino acid stretch insertion reporter constructs with indicated insertions in HEK293 Tet-off cells. Timepoints represent time elapsed after shutoff of transcription shutoff doxycycline. t½ = half-life (min) ± standard error (n = 4). *CRIP1* + SH vs. VI difference in means = 127 min; *p* = 0.01 (two-tailed t-test). *LSM8* + SH vs. VI difference in means = 96 min; *p* = 0.007 (two-tailed t-test). *SPTSSA* + SH vs. VI difference in means = 145 min; *p* = 0.02 (two-tailed t-test). See also S2 Fig for northern blot loading controls, S1 Table for northern probe sequences and reporter ORF sequences, S2 Table for AASC values and statistical data, S3 Table for half-life data and coding sequence sources, and S4 Table for homopolymeric amino acid repeat half-life data.

Upon closer scrutiny of our codon stability coefficients generated for both HeLa and CHO cells, we noted that synonymous codons tended to have similar effects on mRNA stability. In HeLa, for example, all six codons encoding serine are significantly destabilizing; conversely, all four codons encoding valine were significantly stabilizing (Fig 2A). We therefore used a metric analogous to the amino acid stabilization coefficient used by Bazzini and colleagues [39] to determine amino acid-specific effects on mRNA stability in both of our mammalian half-life datasets, defined as the Pearson correlation coefficient between amino acid frequency and half-life (hereafter referred to as AASC; Fig 3A and 3B). In our HeLa dataset, we observed that 9/20 (45%) of amino acids were significantly enriched in stable transcripts (with 7/20 reaching genome-wide significance), whereas 7/20 (35%) of amino acids were significantly enriched in unstable transcripts (with 7/20 reaching genome-wide significance). Similarly, calculation of amino acid stabilization coefficients in CHO cells demonstrated significant enrichment of 9/20 (45%) amino acids in stable transcripts (with 7/20 reaching genome-wide significance) and 8/20 (40%) in unstable transcripts (with 5/20 reaching genome-wide significance). Amino acid-specific effects on stability were also markedly similar in these mammalian cell lines, where 15/20 (75%) of amino acids were stabilizing or destabilizing in both HeLa and CHO cells (S2A Fig; *R* = 0.91, *p* = 3.1 x 10^−8^, Pearson correlation test).

In support of the strong effects of valine and serine frequency on global mRNA stability, we observed that valine frequency (Fig 3C) and serine frequency (Fig 3D) are sufficient to stratify mRNA half-lives in HeLa (*p* < 2.2 x 10^−16^ for both; Kruskal-Wallis test). To further investigate the effects of amino acid content on mRNA half-lives, we utilized a list of human genes previously found to contain naturally occurring homopolymeric repeats of five or more consecutive amino acid residues [57]. We then compared half-lives of genes containing consecutive amino acid repeats of the four most stabilizing amino acids (valine, isoleucine, tyrosine, and alanine; maximum repeat length = 19, median length = 6) versus the four most destabilizing amino acids (serine, histidine, glutamine, and cysteine; maximum repeat length = 58, median length = 6). Consistent with our AASC measurements, transcripts containing stabilizing amino acid repeat stretches showed significantly higher half-lives than transcripts containing destabilizing amino acid repeat stretches (Fig 3E, S4 Table; *p* = 1.1 x 10^−5^, Wilcoxon test).

To expand upon these findings and directly demonstrate the effects of amino acid content on mRNA stability, we designed three sets of reporters based on the endogenous ORF sequence for three small human genes (*CRIP1*, *LSM8*, and *SPTSSA*; average length = 262 nt). We inserted a stretch of 15 amino acids consisting of 5 tandem repeats of either stabilizing amino acids (valine, isoleucine) or destabilizing amino acids (serine, histidine) in the center of each ORF (Fig 3F, S1 Table). To assist with mitigating GC content extrema, codons with more neutral effects on stability were added to each tandem repeat unit—leucine was included after each V+I repeat unit within the “stabilizing” stretch, whereas glutamate was included in the “destabilizing” S+H stretch. Overall, these stretch insertions resulted in an approximately 15% change in amino acid content for each gene. Importantly, inclusion of these neutralizing codons also resulted in 53% optimal codons in both stretches (as defined by tRNA sequencing-based HEK293T tAIs), thereby mitigating confounding effects on stability due to skewed optimal codon content and enabling direct evaluation of amino acid-based effects.

We transfected amino acid stretch reporters into HEK293 Tet-off cells, shut off transcription by doxycycline treatment, and performed mRNA decay analysis by agarose northern blot as above. Strikingly, the reporter mRNA containing the stabilizing VI amino acid stretch insertion was stabilized approximately 2-fold compared to the reporter containing the destabilizing SH stretch in all three gene contexts (Fig 3G, S2B Fig; *CRIP1 p* = 0.01, *LSM8 p* = 0.007, *SPTSSA p* = 0.02, two-tailed t-test), showing that altering amino acid content within human ORFs is sufficient to alter mRNA half-life in human cells, particularly for amino acids with extreme AASCs.

A recent study suggested that transcripts with differential codon content also show differences in polyA tail length, where mRNAs containing a higher proportion of optimal codons also have longer polyA tails [41]. To assess whether the differences in decay rates between our amino acid stretch reporters are accompanied by changes in overall deadenylation, we examined polyA tail length over the transcription shutoff period for the *SPTSSA* reporters containing destabilizing (SH) or stabilizing amino acids (VI) using high-resolution polyacrylamide northern blots. We observed that the reporter containing the stabilizing amino acid stretch had a longer polyA tail length than the reporter containing the destabilizing amino acid stretch at steady-state (0 hr; S2C Fig). Further, the persistence of full-length polyA tails mirrored the calculated half-life for each reporter—the reporter containing the stabilizing stretch showed a higher proportion of full-length polyA tails over the course of the transcription shutoff experiment (S2C Fig). While these results suggest that destabilizing amino acids trigger deadenylation similar to destabilizing codons, it is unclear whether deadenylation is generally rate-limiting for decay of these transcripts.

To further contextualize these results, we returned to our previously published *S*. *cerevisiae* half-life data [28] and calculated AASCs (S3A Fig). We observed a general flattening of AASCs versus our HeLa data, where only 3/20 (15%) of amino acids were significantly associated with stable transcripts and 7/20 (35%) of amino acids were significantly associated with unstable transcripts, albeit with generally smaller effect sizes than observed in our HeLa dataset. Intriguingly, alanine frequency was sufficient to stratify *S*. *cerevisiae* half-lives (S3B Fig; *p* < 2.2 x 10^−16^, Kruskal-Wallis test), and arginine frequency had mild yet significant effects on half-life (S3C Fig; *p* = 8.5 x 10^−12^, Kruskal-Wallis test).

### Codon-specific effects on stability are partially explained by tRNA levels or amino acid levels

**Fig 4:**
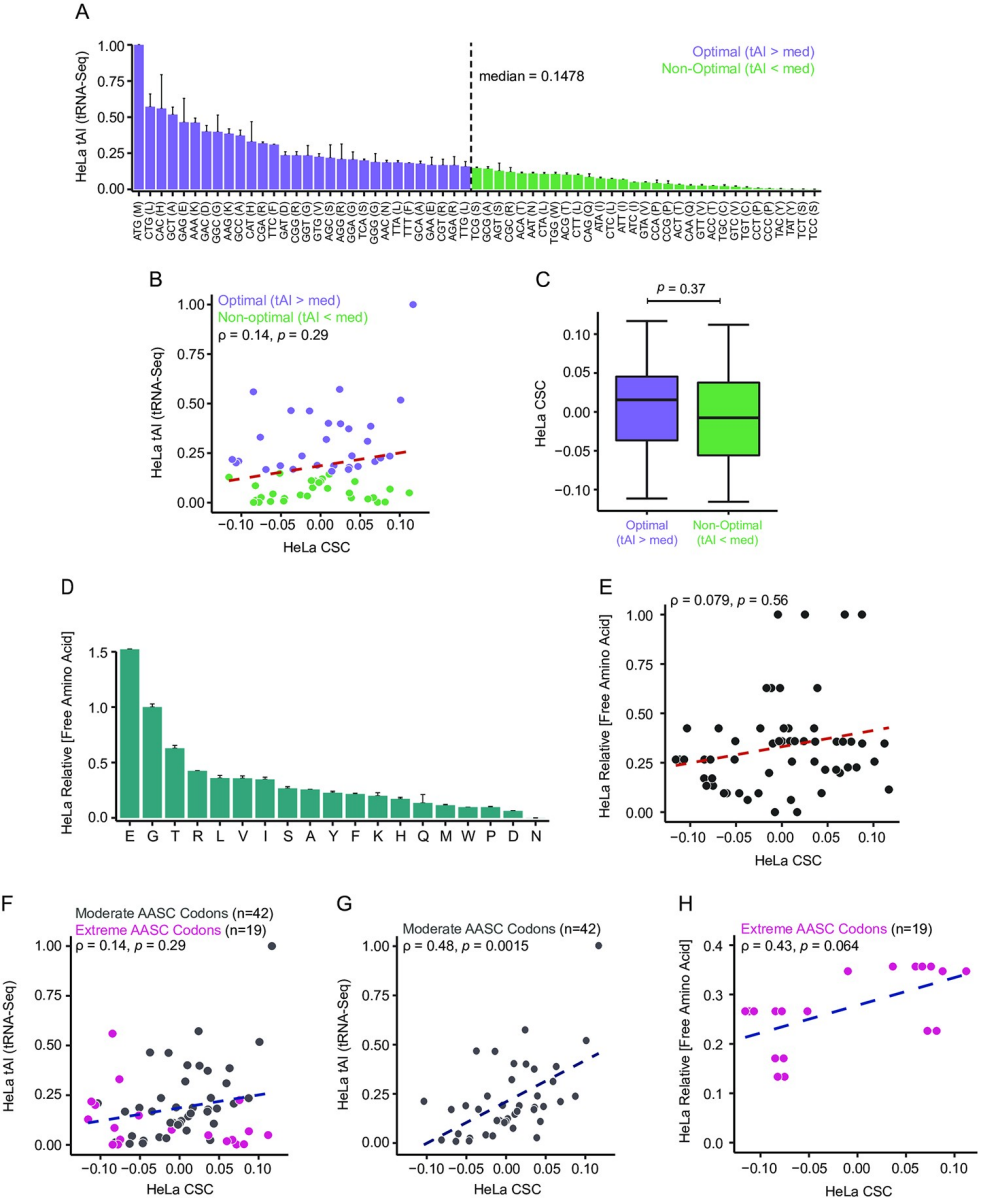
Codon-specific effects on stability are associated with tRNA and amino acid levels. (A) Plot of tRNA adaptive indices (tAI) representing relative tRNA levels calculated from tRNA sequencing data in HeLa cells. Purple represents optimal codons, defined by tAI > median (0.1478). Green represents non-optimal codons, defined by tAI < median. Errorbars indicate standard deviation of three replicates. (B) Plot of HeLa tAI vs. HeLa CSC for all 61 codons. Purple represents optimal codons; green represents non-optimal codons (defined in Fig 4A). Red dotted line indicates linear regression trendline. rho (ρ) = 0.14, *p* = 0.29 (Spearman correlation test). (C) Boxplots comparing distribution of HeLa CSCs for optimal and non-optimal codons as defined in Fig 4A; *p* = 0.37 (Wilcoxon test). (D) Plot of relative free amino acid concentrations (versus glycine) determined from HeLa cell lysates. Errorbars indicate standard deviation of two replicates. (E) Plot of HeLa relative free amino acid concentration vs. HeLa CSC for all codons except those encoding Cys and Glu (n = 57). Red line indicates linear regression trendline. rho (ρ) = 0.079, *p* = 0.56 (Spearman correlation test). (F) Plot of HeLa tAI vs. HeLa CSC for all 61 codons. Blue dotted line indicates linear regression trendline. Gray indicates codons encoding amino acids with mild-moderate effect on stability; magenta indicates codons encoding amino acids with extreme effects on stability (AASC > 0.10 or < -0.10 and *p* < 1.0 x 10^−30^). Overall rho (ρ) = 0.14, *p* = 0.29 (Spearman correlation test). (G) Plot of HeLa tAI vs. HeLa CSC for the 42 codons encoding amino acids with mild-moderate effect on stability. Blue dotted line indicates linear regression trendline. rho (ρ) = 0.48, *p* = 0.0015 (Spearman correlation test). (H) Plot of HeLa relative intracellular amino acid concentration vs. HeLa CSC for the 19 codons encoding amino acids with extreme effects on stability as defined in Fig 4F. Blue dotted line indicates linear regression trendline. rho (ρ) = 0.43, *p* = 0.064 (Spearman correlation test). See also S2 Table for CSC, tAI, and free amino acid concentration values and statistical data.

Previous studies in *S*. *cerevisiae* have shown that tRNA levels explain a large amount of the observed variation in codon stability coefficients, where highly stabilizing codons have high underlying cognate tRNA levels [28,29]. Recent reports have suggested that improved measurement of tRNA levels in human cells will likely demonstrate similar findings [41]. We therefore utilized tRNA-Seq to robustly measure tRNA levels in HeLa cells and calculated tRNA adaptive indices (tAI) as previously calculated for HEK293T tRNA-seq above to estimate overall tRNA availability for each codon (Fig 4A; S2 Table). Using this metric, we defined “optimal” codons as those with tAI greater than the median (tAI = 0.1478) and “non-optimal” codons as those with tAI less than the median. As with HEK293 tAIs, we noted that CG3 codons were not significantly more likely to be designated as optimal by this metric (S4A Fig; *p* = 0.7997, Fisher’s exact test). Globally, HeLa optimal codon content correlated poorly with transcript half-lives (*R* = 0.053, *p* = 8.4 x 10^−9^, Pearson correlation test), but half-lives were generally found to increase with increasing optimal codon content, particularly for transcripts with greater than 70% optimal codons (S4B Fig; *p* < 2.2 x 10^−16^, Kruskal-Wallis test). This result suggests a weaker relationship between tRNA levels and codon-specific stability in HeLa cells versus previously demonstrated effects in *S*. *cerevisiae* [28].

To further investigate the extent to which tRNA levels impact codon-specific effects on stability in human cells, we correlated HeLa tAI levels to our previously calculated HeLa CSC metric. Surprisingly, we observed that the association between tAI and CSC was not significant overall (Fig 4B; rho (ρ) = 0.14, *p* = 0.29, Spearman correlation test); indeed, codon optimality as defined by tRNA-seq was not significantly predictive of CSC in HeLa cells (Fig 4C; median CSC for optimal codons = 0.0154, median CSC for non-optimal codons = -0.0075, *p* = 0.37, Wilcoxon test). To explore other potential contributing factors underlying differences in codon-specific effects on stability, we next measured free intracellular amino acid levels in HeLa cells by reverse phase HPLC (Fig 4D). Due to technical limitations, this assay was not able to detect cysteine levels. We also observed very high glutamate levels, likely driven by the abnormal usage of glutamine and glutamate as alternative carbon sources in cancer cells, including HeLa [58,59]. Therefore, we excluded codons encoding glutamate (n = 2) and cysteine (n = 2) from downstream analyses. Overall, free amino acid levels were also not significantly associated with codon stability coefficients (Fig 4E; rho (ρ) = 0.079, *p* = 0.56, Spearman correlation test).

To determine whether a combination of tRNA levels and amino acid concentrations is more predictive of CSC values than either variable alone, we generated a joint linear regression model of HeLa CSC values using HeLa tAI and free amino acid levels (Table 1). In agreement with our individual assessments of possible associations with CSC, this joint regression model of tAI and free amino acid concentrations did not demonstrate significant predictive value in determining HeLa CSCs (overall model *p* = 0.0962). However, in light of our findings that amino acid content can also contribute to mRNA stability and the observation that the association between tAI and CSC was much weaker when considering CSCs calculated from frameshifted ORFs (S4C Fig), we wondered whether underlying amino acid effects could be masking the relationship between tRNA levels and codon-based effects on stability. Strikingly, separation of codons encoding the three amino acids with extreme stabilizing effects (Val, Ile, Tyr; AASC > 0.10, *p* < 1.0 x 10^−30^) and the three amino acids with extreme destabilizing effects (Ser, His, Gln; AASC < -0.10, *p* < 1.0 x 10^−30^) from the other 14 amino acids with more mild stabilizing or destabilizing effects revealed a more robust association between tRNA availability and CSC for the 42 codons with more moderate underlying amino acid effects (Fig 4F and 4G; rho (ρ) = 0.48, *p* = 0.0015, Spearman correlation test). Conversely, CSCs for codons encoding amino acids with extreme AASCs were uncoupled from underlying tRNA levels (Fig 4F; rho (ρ) = -0.34, *p* = 0.15, Spearman correlation test), suggesting that the effects on stability for some codons are associated with underlying amino acid effects in addition to cognate tRNA concentration. Importantly, these associations appeared to be frame-dependent, where evaluation of the relationship between tAI and CSCs calculated from frameshifted ORFs demonstrated a weaker association with tRNA levels (S4D Fig). Accordingly, joint regression of tRNA and free amino acid levels for the 38 codons encoding amino acids with moderate effects on stability demonstrated that only tAI was significantly predictive of CSC for these codons (Table 1; overall model *p* = 0.001).

**Table 1:** tRNA and free amino acid levels are associated with CSC values based on underlying amino acid effects. Summary of outputs for linear models of HeLa CSC using indicated variables (formula: CSC ~ HeLa tAI + free amino acid concentration). Codons encoding Cys and Glu were excluded based on NA or extreme outlier status from intracellular amino acid determinations. β estimates denote predicted effect on CSC with a linear increase of the indicated variable. 95% CI = confidence interval for β estimates.

**All codons (n = 57)**				
	**β Estimate**	**95% CI**		**P-value**
(Intercept)	-0.0286	-0.0611	0.0038	0.0826
HeLa tAI	0.0736	-0.0143	0.1615	0.099
[Free amino acid]	0.0507	-0.0171	0.1185	0.1399
**Adjusted R-squared**	0.0491	**Overall P-value**		0.0962
**Moderate amino acid effect codons (n = 38)**				
	**β Estimate**	**95% CI**		**P-value**
(Intercept)	-0.0293	-0.0566	-0.0021	0.0355
HeLa tAI	0.137	0.0677	0.2064	0.0003
[Free amino acid]	0.0257	-0.0217	0.0731	0.2788
**Adjusted R-squared**	0.2849	**Overall P-value**		0.001
**Extreme amino acid effect codons (n = 19)**				
	**β Estimate**	**95% CI**		**P-value**
(Intercept)	0.2901	-0.2865	-0.0075	0.0401
HeLa tAI	-0.1351	-0.3849	0.1148	0.2686
[Free amino acid]	0.5478	0.0903	1.0053	0.0219
**Adjusted R-squared**	0.326	**Overall P-value**		0.0166

In contrast, separate consideration of codons with the six strongest underlying AASCs showed a stronger (yet statistically insignificant) association with free amino acid levels (Fig 4H; rho (ρ) = 0.43, *p* = 0.064, Spearman correlation test), whereas codons with more moderate underlying amino acid effects were not associated with free amino acid levels (rho (ρ) = 0.00077, *p* = 1, Spearman correlation test); however, it is important to note that repeated usage of the same free amino acid level metric for synonymous codons may violate the assumption of independence for the purposes of this analysis. As with tRNA levels, the putative association between free amino acid levels and CSCs for codons with strong underlying amino acid effects was found to be substantially weakened for out-of-frame CSCs (S4E Fig). Taken together, these data suggest that codon-specific effects on stability may be influenced by underlying tRNA levels; however, codons with strong underlying amino acid-based effects on stability may be influenced by intracellular amino acid levels. Accordingly, joint regression of tRNA and free amino acid levels for the 19 codons encoding amino acids with extreme effects on stability demonstrated that only free amino acid levels were significantly predictive of CSC for these codons (Table 1; overall model *p* = 0.0166).

### Both codon and amino acid content contribute to mRNA stability transcriptome-wide

We next sought to build a linear model to evaluate the contribution of codon and amino acid content to transcriptome-wide half-lives in HeLa cells. Due to the right skew evident in our HeLa half-life dataset, we first utilized a reciprocal transformation (-1/x) to make our half-life data more closely resemble a standard normal distribution suitable for linear modeling (S5A Fig). To represent overall levels of stabilizing versus destabilizing codons in a given ORF sequence, we calculated a transcript-level average CSC, where increasing transcript-level CSC values indicate increasing usage of stabilizing codons. Similarly, we also calculated transcript-level AASCs to represent overall stabilizing versus destabilizing amino acid usage for a given ORF sequence. Based on recent evidence suggesting that codons with G or C at the 3’-position (GC3 codons) and overall GC content are significant predictors of transcript stability [43], we also considered GC3 codon frequency and GC content in our half-life model. We noted that all four of these variables were approximately normally distributed in our HeLa half-life dataset; however, both overall GC content and GC3 codon frequency showed distinctive bimodal distributions (S5B Fig).

Upon evaluating the association between each of these four variables and HeLa half-lives in isolation, we observed that average CSC and average AASC generally show a stronger association with half-life values than either GC3 codon frequency or GC content (S5C Fig). We further noted a high correlation between transcript-level CSCs and AASCs (S5C Fig; *R* = 0.938, *p* < 2.2 x 10^−16^, Pearson correlation test), suggesting that codon- and amino acid-specific contributions to mRNA stability may be highly interconnected. Similarly, GC3 codon frequency and overall GC content also appear to demonstrate covariation (S5C Fig; *R* = 0.932, *p* < 2.2 x 10^−16^, Pearson correlation test). We therefore created a joint linear regression model of transformed HeLa half-lives using only average CSC and GC3 codon frequency (Table 2).

**Table 2:** Overall codon content is more predictive of HeLa mRNA half-lives than GC3 codon frequency. Summary of output for linear model of -1/half-life for 11,800 HeLa mRNAs using indicated variables (formula: -1/Half-life ~ Average CSC + GC3 codon frequency). β estimates indicate predicted effect on -1/half-life value with a linear increase of the indicated variable. 95% CI = confidence interval for β estimates.

	β Estimate	95% CI		P-value
(Intercept)	-0.2901	-0.2953	-0.2848	< 2.2 x 10^−16^
Transcript Average CSC	4.2985	4.1034	4.4935	< 2.2 x 10^−16^
GC3 Codon Frequency	-0.0201	-0.0288	-0.0113	7.48 x 10^−6^
**Adjusted R-squared**	0.1368	**Overall P-value**		< 2.2 x 10^−16^

Based on the results of our linear regression analysis, average codon composition (represented by transcript average CSC) and GC3 codon frequency together account for approximately 13.7% of variation in half-lives transcriptome-wide (adjusted R-squared = 0.1368; Table 2), where increasing average CSC values are significantly associated with increasing half-life values (Table 2; β = 4.30, *p* < 2.2 x 10^−16^). In contrast, GC3 codon frequency is less predictive of half-lives; indeed, increasing GC3 codon frequency is significantly associated with a slight decrease in half-life (Table 2; β = -0.02, *p* = 7.48 x 10^−6^). Accordingly, we noted that increasing transcript-level CSC values are associated with a linear increase in half-life in our HeLa dataset, whereas increasing GC3 codon frequencies result in relatively constant HeLa half-lives (S5D Fig).

**Fig 5:**
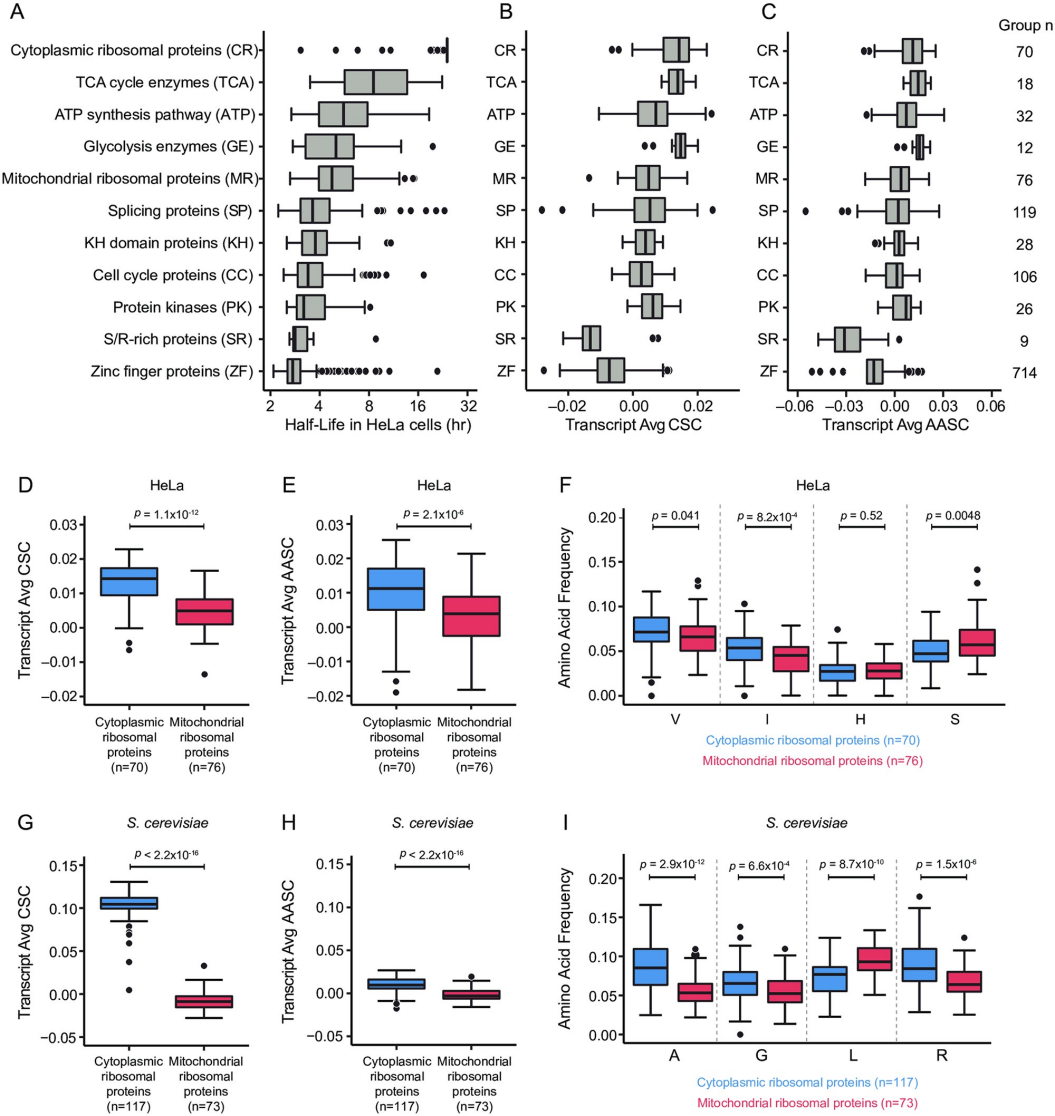
Codon and amino acid content are associated with observed differences in mRNA stability among functionally related genes. (A) Boxplots comparing endogenous HeLa mRNA half-life distributions for indicated gene groups. Overall difference in means among all groups *p* < 2.2 x 10^−16^ (Kruskal-Wallis test). (B) Boxplots comparing transcript average codon stability coefficient (CSC) distributions for indicated gene groups. Overall half-life vs. transcript average CSC *R* = 0.5413; *p* < 2.2 x 10^−16^ (Pearson correlation test). (C) Boxplots comparing transcript average amino acid stabilization coefficient (AASC) distributions for indicated gene groups. Overall half-life vs. transcript average AASC *R* = 0.4626; *p* < 2.2 x 10^−16^ (Pearson correlation test). (D) Boxplots directly comparing transcript average CSC distributions for human cytoplasmic ribosomal proteins (blue) and mitochondrial ribosomal proteins (red) using HeLa CSC values; *p* = 1.1 x 10^−12^ (Wilcoxon test). (E) Boxplots directly comparing transcript average AASC distributions for human cytoplasmic ribosomal proteins (blue) and mitochondrial ribosomal proteins (red) using HeLa AASC values; *p* = 2.1 x 10^−6^ (Wilcoxon test). (F) Boxplots directly comparing amino acid frequency distributions for indicated stabilizing (V, I) and destabilizing (H, S) amino acids (defined by HeLa AASC) for human cytoplasmic ribosomal proteins (blue) and mitochondrial ribosomal proteins (red). P-values indicate results of Wilcoxon test of difference in means for each amino acid. (G) Boxplots directly comparing transcript average CSC distributions for *S*. *cerevisiae* cytoplasmic ribosomal proteins (blue) and mitochondrial ribosomal proteins (red) using *S*. *cerevisiae* CSC values; *p* < 2.2 x 10^−16^ (Wilcoxon test). (H) Boxplots directly comparing transcript average AASC distributions for *S*. *cerevisiae* cytoplasmic ribosomal proteins (blue) and mitochondrial ribosomal proteins (red) using *S*. *cerevisiae* AASC values; *p* < 2.2 x 10^−16^ (Wilcoxon test). (I) Boxplots directly comparing amino acid frequency distributions for indicated stabilizing (A, G) and destabilizing (L, R) amino acids (defined by *S*. *cerevisiae* AASC) for *S*. *cerevisiae* cytoplasmic ribosomal proteins (blue) and mitochondrial ribosomal proteins (red). P-values indicate results of Wilcoxon test of difference in means for each amino acid. See also S2 Table for CSC and AASC values and S5 Table for gene group designations, half-life data, and average CSC/AASC values for HeLa and *S*. *cerevisiae*.

To further narrow our evaluation of the impact of codon and amino acid content on endogenous mRNA half-lives, we examined several functionally related gene groups that tended to display similar half-lives in our HeLa endogenous mRNA half-life dataset, suggesting that these genes may be coordinately controlled at the level of mRNA stability (Fig 5A, S5A Table). For example, genes involved in key metabolic pathways tended to display relatively high mRNA half-lives (e.g. TCA cycle enzyme group median half-life = 8.53 hrs); conversely, genes involved in regulatory pathways tended to be more unstable (e.g. Serine/Arginine-rich (SR) protein group median half-life = 2.83 hrs). We reasoned that the half-lives for these gene groups may be more subject to coordinated control via synonymous codon and amino acid content within the open reading frame.

To examine the relationship between codon usage and mRNA half-life in these gene groups, we first examined transcript-level CSCs for each transcript. Average CSC values were significantly associated with mRNA half-life across all gene groups examined (Fig 5B; *R* = 0.5413; *p* < 2.2 x 10^−16^); further, gene groups with high mRNA stability also tended to have higher transcript-level CSCs (e.g. TCA cycle enzyme group median CSC = 0.0139), while gene groups with relatively low mRNA stability showed lower transcript-level CSCs (e.g. SR protein group median CSC = −0.0132). Analysis of transcript-level AASCs similarly showed a significant association between mRNA half-life and transcript-level AASC (Fig 5C; *R* = 0.4626; *p* < 2.2 x 10^−16^, Pearson correlation test), where more stable gene groups tended to show higher transcript-level AASCs (e.g. TCA cycle enzyme group median AASC = 0.0145) and less stable gene groups had lower transcript-level AASC values (e.g. SR protein group median AASC = −0.0311).

Given that transcript-level CSC and AASC values appear to be associated with half-life transcriptome-wide and across these gene groups overall, we further focused on the effects of codon and amino acid content on overall mRNA half-life by considering two gene groups of interest, the cytoplasmic and mitochondrial ribosomal proteins. While these gene groups serve similar functions, they show differences in amino acid sequence composition and conservation—specifically, cytoplasmic ribosomal proteins show much higher levels of sequence conservation between rats and yeast, whereas mitochondrial ribosomal proteins show low sequence similarity between species [60,61]. Interestingly, cytoplasmic ribosomal proteins are also markedly more stable as a class (Fig 5A; cytoplasmic ribosomal protein median half-life = 24 hrs vs. mitochondrial ribosomal protein median half-life = 4.75 hrs; *p* = 0.0009, Wilcoxon test). Similarly, cytoplasmic ribosomal proteins have significantly higher transcript-level CSCs (Fig 5D; *p* = 1.1 x 10^−12^, Wilcoxon test) and AASCs (Fig 5E; *p* = 2.1 x 10^−6^, Wilcoxon test), indicating overall increased usage of stabilizing codons and amino acids versus mitochondrial ribosomal proteins. Further analysis of individual amino acid frequencies recapitulated transcript-level AASC differences between the ribosomal protein groups (Fig 5F), where cytoplasmic ribosomal proteins were found to contain significantly more stabilizing amino acids (such as valine and isoleucine) and significantly fewer destabilizing amino acids (such as serine) than mitochondrial ribosomal proteins. Together, these observations suggest that the observed differences in half-lives between the cytoplasmic and mitochondrial ribosomal proteins may be due in part to differences in ORF sequence composition.

To continue our assessment of the relative contributions of codon and amino acid-based effects on mRNA half-life, we turned again to our previously published *S*. *cerevisiae* data [28] to compare transcript average CSC and AASC values between the cytoplasmic and mitochondrial ribosomal proteins (S5B Table). Consistent with the dominance of codon-based effects in this system, we observed a large, highly significant difference in transcript-level CSC values between the cytoplasmic and mitochondrial ribosomal proteins (Fig 5G; *p* < 2.2 x 10^−16^, Wilcoxon test). Surprisingly, we noted that average AASC also differed among these groups, albeit with a smaller effect size (Fig 5H; *p* < 2.2 x 10^−16^, Wilcoxon test); similarly, amino acid frequencies for amino acids identified as stabilizing (such as alanine and glycine) were significantly higher in the cytoplasmic ribosomal proteins (Fig 5I), but frequencies of amino acids found to be destabilizing (such as leucine and arginine) were not uniformly lower in cytoplasmic ribosomal proteins, consistent with weaker underlying amino acid effects.

Taken together, these analyses of half-lives transcriptome-wide and in functionally related gene groups support the idea that synonymous codon and amino acid content partially contribute to observed differences in mRNA half-lives in human cells, suggesting that both of these factors may play an important role in determining overall mRNA expression levels.

### Codon- and amino acid-based effects on mRNA stability vary among different species

**Fig 6:**
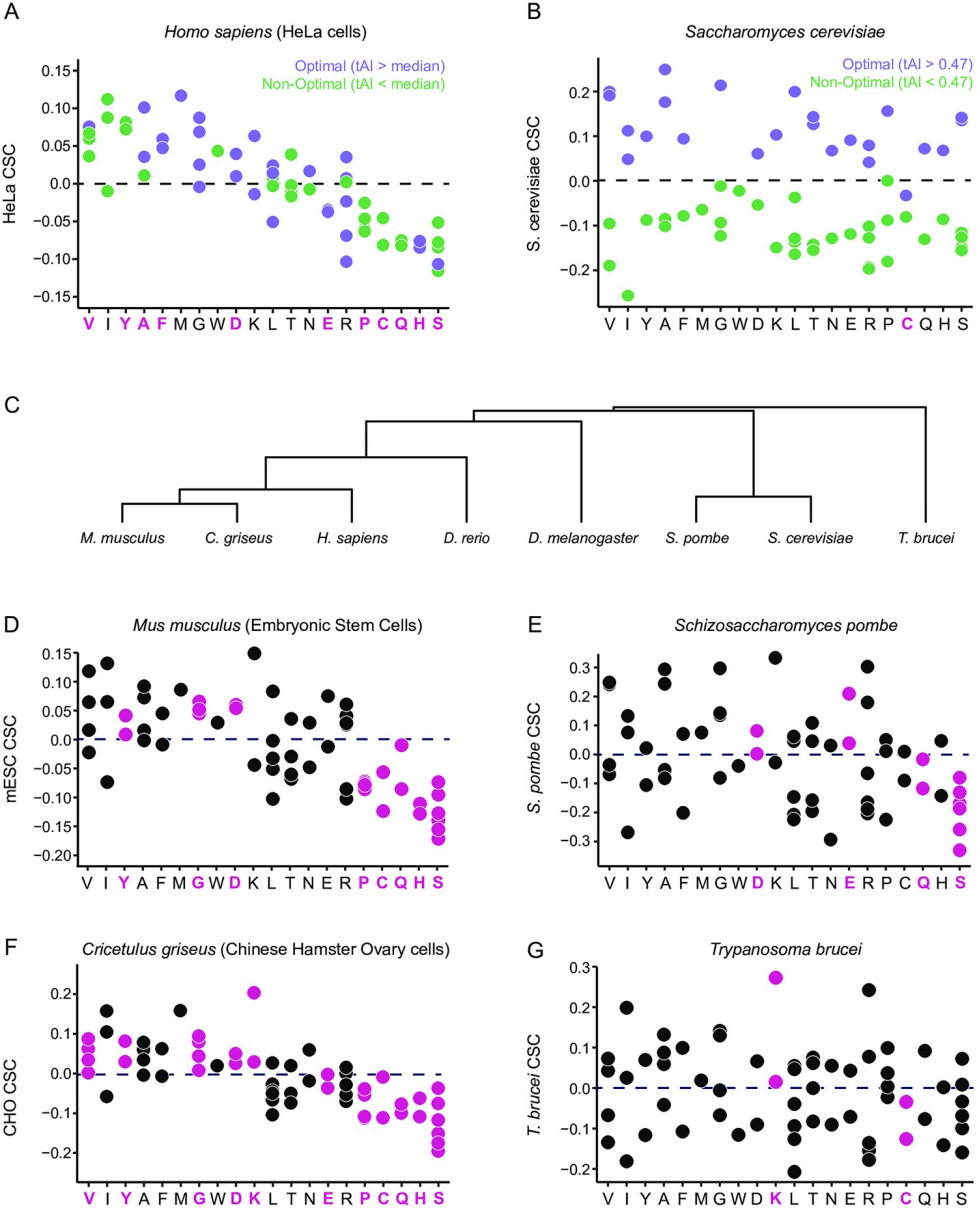
Codon and amino acid-based effects on mRNA stability differ between mammals and yeast. (A) Plot of codon stability coefficients (CSC) grouped by encoded amino acid for endogenous HeLa mRNAs. Purple indicates optimal codons; green indicates non-optimal codons (defined by tRNA sequencing-based tRNA adaptive indices in Fig 4A). Amino acids with exclusively stabilizing (CSC > 0) or destabilizing (CSC < 0) synonymous codons are indicated by bolded magenta letters (n = 11). (B) Plot of codon stability coefficients (CSC) grouped by encoded amino acid for *S*. *cerevisiae* (budding yeast) mRNAs [28]. Amino acids with exclusively stabilizing (CSC > 0) or destabilizing (CSC < 0) synonymous codons are indicated by bolded magenta letters (n = 1). Purple indicates optimal codons; green indicates non-optimal codons (defined by tRNA adaptive indices in [28]). (C) Phylogenetic tree diagram showing qualitative evolutionary relationships between the 8 species presented in Fig 6A-G and S6A and S6B Fig, generated using the Interactive Tree of Life (iTOL) online tool [98]. (D-G) Plots of codon stability coefficients (CSC) grouped by encoded amino acid for (D) *Mus musculus* (embryonic stem cells), (E) *Schizosaccharomyces pombe* (fission yeast), (F) *Cricetulus griseus* (Chinese hamster ovary cells), and (G) *Trypanosoma brucei* (bloodstream lifecycle stage). Magenta indicates amino acids encoded exclusively by stabilizing (CSC > 0) or destabilizing (CSC < 0) synonymous codons. See also S2 Table for CSC values and S3 Table for half-life data sources.

Collectively, our analyses of the relative effects of specific codons and amino acids on mRNA stability in mammalian cells suggest that codon- and amino acid-specific effects on mRNA stability are closely linked, as evidenced by clustering of codon stability coefficients by amino acid in HeLa (Fig 6A)—specifically, 11/18 (61%) of the amino acids with two or more synonymous codons showed exclusively stabilizing or destabilizing effects (all synonymous codons CSC > 0 or CSC < 0, respectively). This finding starkly contrasts with our previous findings in *S*. *cerevisiae*, where synonymous codons tend to have either stabilizing or destabilizing effects [28]. Indeed, in *S*. *cerevisiae*, all amino acids with two or more synonymous codons (except cysteine) have clear differential effects on stability (Fig 6B).

To expand upon these findings, we examined relative contributions of codon and amino acid-based effects on stability in other species by mining previously published half-life datasets for several representative organisms (Fig 6C–6G, S6A and S6B Fig). These organisms represent the wide evolutionary space between humans and budding yeast, including two additional mammals (*Cricetulus griseus* and *Mus musculus*), two more distantly related metazoans (*Drosophila melanogaster* and *Danio rerio*), a protist (*Trypanosoma brucei*), and an additional fungus (*Schizosaccharomyces pombe*). We calculated CSCs for each dataset and binned them by encoded amino acid to qualitatively evaluate differences in codon- and amino acid-mediated effects on stability. Intriguingly, the two other mammals showed highly similar patterning to HeLa cells, where CSCs were generally clustered by amino acid (8/18 and 11/18 amino acids exclusively stabilizing/destabilizing in *M*. *musculus* and *C*. *griseus*, respectively; Fig 6D and 6F). Similarly, two other metazoan species showed some limited spread in CSC between synonymous codons (9/18 and 6/18 amino acids exclusively stabilizing/destabilizing in *D*. *rerio* and *D*. *melanogaster*, respectively; S6A and S6B Fig). In contrast, *S*. *pombe* and *T*. *brucei* more closely resemble *S*. *cerevisiae*, with wide CSC variation among synonymous codons for the majority of amino acids (4/18 and 2/18 amino acids exclusively stabilizing/destabilizing in *S*. *pombe* and *T*. *brucei*, respectively; Fig 6E and 6G). While these findings may be more broadly indicative of differences in evolutionary constraints on synonymous codon usage [62,63], they may also reflect important evolutionary differences in underlying mRNA decay mechanisms, representing a shift from predominantly codon-specific effects on stability in eukaryotes such as yeast to more prominent underlying amino acid effects in higher eukaryotes.

## Discussion

In this study, we demonstrate that coding region determinants have an impact on mammalian mRNA half-lives. First, we investigate the effects of optimal codon content on mRNA half-life in the context of naturally occurring human ORFs, demonstrating that changing synonymous codon content is sufficient to alter human mRNA half-lives (Fig 1). Further, we expand upon recent work showing global codon-specific effects on mRNA stability in human cells and describe similar effects in an additional mammalian system, Chinese hamster ovary cells (Fig 2). Surprisingly, global analysis also shows that enrichment of specific amino acids in mammalian ORFs results in appreciable effects on mRNA stability. In support of this observation, we provide evidence that adding a small number of extreme stabilizing or destabilizing amino acids to an mRNA is sufficient to affect its half-life (Fig 3). Both codon and amino acid content are significantly associated with observed half-lives for functionally related human mRNAs, further confirming the coding sequence itself as a determinant of mRNA stability (Fig 5) [41,42,64,65]. In addition to known 3’-UTR-based effects on mRNA stability such as miRNA binding sites and destabilizing AU-rich elements [6,20,66], our findings point to coding sequence as an important determinant of mammalian mRNA half-lives. Of note, previous studies in *S*. *cerevisiae* have demonstrated that single synonymous mutations can result in mRNA destabilization in specific sequence contexts [67]. Moving forward, it will be of interest to determine whether single synonymous codon variations can impact mRNA stability in mammalian genes, with direct implications for understanding how such “silent” mutations can result in human disease.

**Fig 7:**
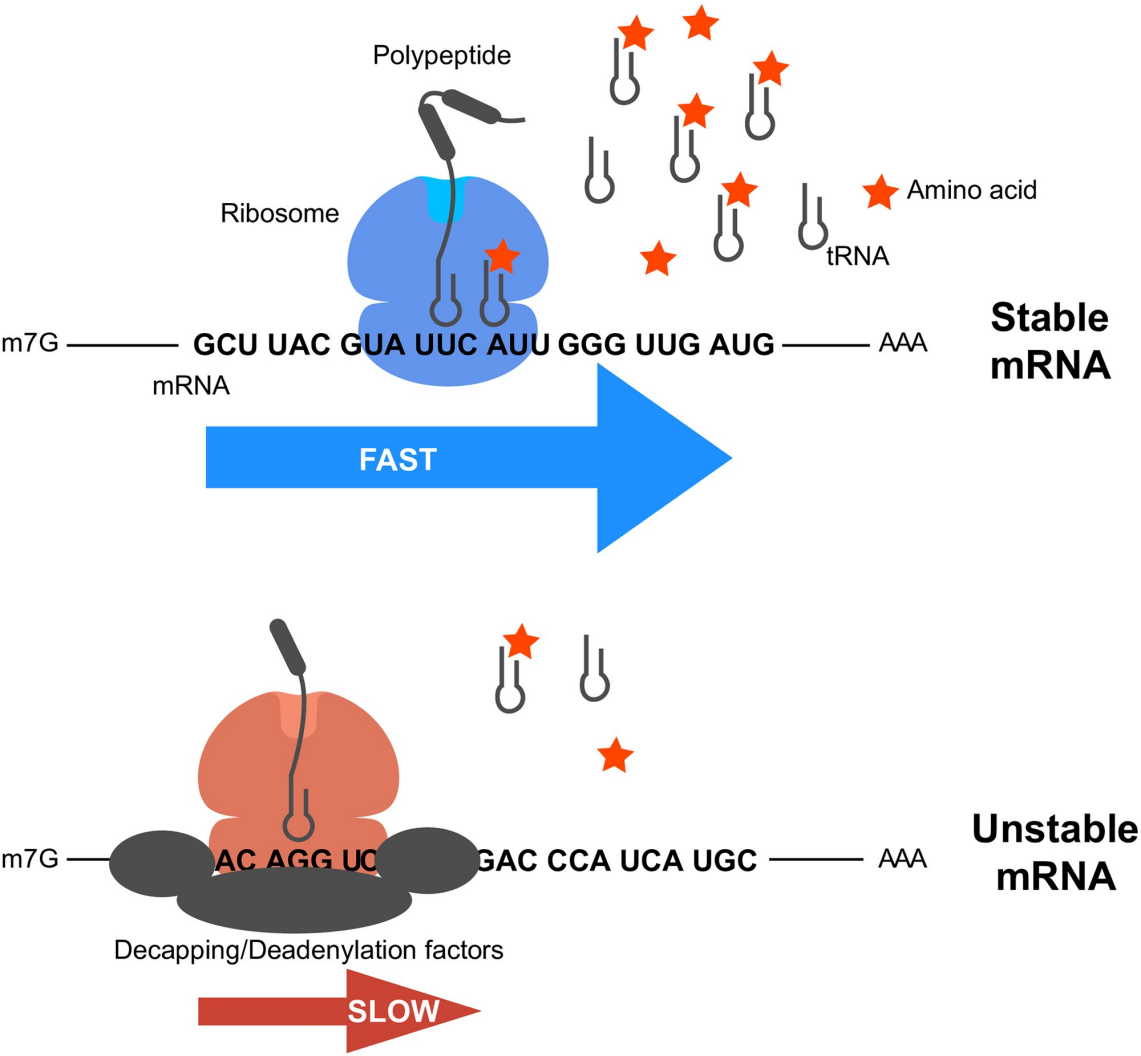
Model of coordinated codon and amino acid-based effects on mRNA stability in mammalian cells. Model of coordinate effects of codon and amino acid content on mRNA stability in mammalian cells. In this model, the stability of a given mRNA ORF sequence is related to functional cognate tRNA availability, which is in turn influenced by upstream amino acid concentrations. Stable mRNAs contain high proportions of codons with relatively high functional tRNA availability and are translated quickly. In contrast, mRNAs containing high proportions of codons with low tRNA availability are translated more slowly and targeted for decay via unknown protein effectors, which ultimately recruit decapping/deadenylation factors.

In an effort to explore the underlying causes that explain differences in codon- and amino acid-specific effects on stability, we determined that both underlying cognate tRNA concentrations and intracellular amino acid levels are associated with stability for different codon subsets—specifically, CSC values for codons without strong underlying amino acid effects appear to be significantly associated with tRNA levels, whereas codons encoding extremely stabilizing or destabilizing amino acids may be influenced by free amino acid levels (Fig 4). Collectively, these data suggest a model where tRNA expression and upstream free amino acid concentrations coordinate functional tRNA pools, thereby affecting translation elongation speed for specific codons and amino acids. These differences are ultimately sensed by the mRNA degradation machinery, where ORFs with high proportions of destabilizing codons and amino acids are targeted for rapid degradation (Fig 7). In support of this model, previous studies in human cells have established that codon-specific effects on mRNA stability in humans are translation-dependent, specifically at the level of translation elongation [41,42]. Translational pausing has also been demonstrated for specific amino acids, particularly proline and charged amino acids [68–72]. Further perturbative studies will be necessary to positively identify and define the contribution of tRNA levels and intracellular amino acid levels to mRNA stability and to refine our understanding of differences in translation over specific codons and amino acids—these efforts may be aided by improved ribosome profiling methods with additional capacity to detect paused ribosomes [68,72].

The strong amino acid-based effects on mRNA stability that we observed in this study raise the possibility of dynamic control of gene expression via mRNA stability under different metabolic states. Indeed, it has been documented that changes in free amino acid levels can regulate gene expression in eukaryotes, including at the level of mRNA stability [73,74]. Especially in the context of cancer, increased shuttling of amino acids to various metabolic processes could ultimately affect their availability for tRNA aminoacylation. For example, serine is not only available for charging tRNA, but is also a key carbon source that feeds into the one-carbon metabolism pathway, which is critical for a number of pathways that support uncontrolled cancer cell growth; these include production of antioxidants, generation of substrates for DNA/RNA methylation, and biosynthesis of phospholipids, other amino acids, and nucleotides [75–77]. Likewise, previous studies have demonstrated that changes in tRNA expression can directly influence stability of selected mRNAs [64]. Recent studies have also delineated numerous examples of extensive dysregulation of tRNA expression, notably in cancer cells [46,78,79]. Interestingly, tRNA levels also change throughout development, during stress, and in various neurological disorders caused by mutations in tRNA biogenesis factors [27,48,80,81]. Moving forward, it will be critical to establish whether the amino acid- and tRNA-based effects on stability we describe here are present in other cell types and investigate how changes in codon- and amino acid-specific stability may vary under various stress conditions and nutritional states.

Ultimately, our findings that both codon and amino acid content are important factors in modulating mammalian mRNA stability may point towards differences in normal mRNA turnover between yeast and mammals. While 5’-3’ deadenylation-dependent decay and 3’-5’ decay by the cytoplasmic exosome have been extensively characterized as the two major mRNA turnover pathways in *S*. *cerevisiae* [3,5,82], there is a considerable gap in our knowledge of general mammalian mRNA turnover pathways. Interestingly, more recent studies in human cells have suggested novel decay mechanisms with extensive contribution to mRNA half-life [8]. Interplay between these different mRNA turnover pathways and *cis*-acting destabilizing elements within the 3’-UTR could potentially mask or modulate underlying ORF-mediated effects on stability in mammalian cells. It will also be critical to identify sensors of ribosome states that trigger mRNA decay, analogous to the role of Dhh1p in sensing differences in translation over optimal and non-optimal codons in *S*. *cerevisiae* [29]. While the Dhh1p human homolog, DDX6, has been implicated in miRNA-mediated decay and other specific decay contexts [83–85], it is currently unclear whether it also plays a role in sensing a preponderance of destabilizing codons in mammalian systems. Consistent with the possibility of differences in downstream decay pathways, recent studies also hint that novel protein effectors may be involved in sensing codon content both globally and in the context of specific promoters [43,65].

The observation that codon-based effects on stability are generally dampened in higher eukaryotes compared to yeast also raises interesting questions about potential differences in mRNA decoding between these organisms. One possible explanation could lie in novel mRNA modifications [86,87] or tRNA modifications at the anticodon loop [88,89] in mammals that act to modulate the strong codon effects seen in yeast. In particular, modifications in the wobble position of tRNAs and mRNAs have been shown to be critical for enhancing translation and promoting fidelity in decoding [56,90–92], potentially indicating a mechanism for enhancing decoding of specific codons independently of cognate tRNA levels. Further studies will be needed to fully characterize the breadth of modifications in the mammalian epitranscriptome and their potential impact on modulating codon optimality.

Collectively, this study highlights both important similarities and critical differences in regulation of mRNA stability at the level of the open reading frame sequence between different species, where both codon- and amino acid-based effects contribute at various levels in different organisms across a wide span of evolutionary time. These differences suggest novel avenues of investigation that will aid in our overall understanding of gene expression regulation in mammalian cells.

## Methods and materials

### Cell lines and growth conditions

Wild type HeLa cells (human cervical adenocarcinoma, ATCC CCL-2) were maintained in DMEM, high glucose, pyruvate, no glutamine (Thermo Fisher Scientific 10313) supplemented with 10% Bovine Calf Serum (BCS; Hyclone SH30073.03) and 4 mM L-Glutamine (Thermo Fisher Scientific 25030149) and grown at 37°C with 5% CO_2_ supplementation. Cells were passaged by trypsinization approximately every 3–5 days.

Chinese hamster ovary cells (CHO-K1, ATCC CCL-61) were maintained in DMEM, high glucose, pyruvate, glutamine (Thermo Fisher Scientific 11995) supplemented with 10% Fetal Bovine Serum (FBS; Gibco 1992275), 1% Penicillin/Streptomycin, and 20 μg/mL L-proline (VWR AAA10199-14) and grown at 37°C with 5% CO_2_ supplementation. Cells were passaged by trypsinization approximately every 3–5 days.

HEK293 Tet-Off^®^ cells (human embryonic kidney, Clontech 631152) were maintained in DMEM, high glucose, pyruvate, glutamine (Thermo Fisher Scientific 11995) supplemented with 10% Fetal Bovine Serum (FBS; Gibco 1992275) and 1% Penicillin/Streptomycin and grown at 37°C with 5% CO_2_ supplementation. Cells were passaged by trypsinization approximately every 3–5 days.

### Plasmids and oligonucleotides

All plasmids (including reporter open reading frame insert sequences) and northern blot probe sequences used in this study are listed in S1 Table.

### Global half-life analysis in CHO cells by 5-EU-Seq

Large scale RNA decay analyses were performed in CHO cells by an integrated approach combining metabolic labeling with 5-ethynyluridine (Thermo Fisher Scientific Click-iT nascent RNA capture kit, C10365; [93]) and next generation sequencing library preparation [94]. Cells were seeded into eleven 60mm dishes and pulse-labelled with 0.2 mM of 5-ethynyluridine (5-EU) at 40% confluency, keeping one plate not labelled for a control without 5-EU. 5-EU was chased after 24 hr of incorporation by adding 5 mM of uridine. Cells were collected by replacing the media by 1 mL Trizol at the following time-points: 0 min, 30 min, 1 hr, 2 hr, 3 hr, 4 hr, 6 hr, 8 hr, 10 hr and 12 hr of chase. RNA was extracted following the Trizol extraction protocol, then re-extracted with phenol-chloroform as in [95]. RNA was precipitated with sodium acetate and 100% ethanol at -20°C, then spun down at 16,000 g for 20 min at 4°C, washed twice with 70% EtOH and resuspended in water. DNAse I treatment was performed on duplicate RNA samples (2 x 6 μg) after addition of 5 ng of in vitro-labelled spike-in to each sample. The spike-in mix consisted of partial RNA sequences of *B*.*subtilis* LYSa and firefly luciferase transcripts, of which the gene sequences were cloned into pBluescript SK+ plasmid (pJC879 and pJC880, respectively) and transcribed using T7 RNA polymerase and 2 mM of 5-EU. Samples were depleted of ribosomal RNAs using Illumina Ribo-Zero Gold rRNA removal kit (MRZG12324), then purified using the RNA Clean up and concentrator Kit (R1015, the epigenetics company). The duplicates for each sample were then combined before RNA fragmentation by alkaline treatment at 95°C for 25 min (NaCO3 pH9.2 50 mM final, EDTA 1 mM final). The reaction was immediately stopped on ice by addition of 0.3 M NaOAc pH5.2, 2μL glycoblue and 500μL water. RNA was precipitated with isopropanol, spun at 16,000 g for 30 min at 4°C, washed with 70% EtOH and resuspended in 15.75 μL of water. Biotinylation of the 5-EU labelled RNA was performed following the Click-iT nascent RNA capture kit protocol and precipitated at -80°C overnight. The fragmented and biotinylated RNA was purified on NuPAGE denaturing polyacrylamide gels (TBE-urea 15% polyacrylamide, Thermo Fisher Scientific EC62152BOX) as in [94], excising RNA between 20 and 70 nucleotides. After extraction from the gel with 500 μL of RNA gel extraction buffer (300 mM NaOAc pH5.2, 1 mM EDTA, 0.25% (w/v) SDS) on a rotator at room temperature overnight, samples were precipitated with isopropanol and resuspended in 10 μL of 10 mM Tris-HCL pH8.

Pulldown of the biotinylated RNA was performed following the Click-iT nascent RNA capture kit protocol, using 50 μL of C1 myOne streptavidin beads (Thermo Fisher Scientific 65001) per reaction. The beads were then resuspended in 20 μL of wash buffer from the kit and the RNA was dephosphorylated on the beads by adding 10 μL of 10 mM Tris-HCL pH8 and 10 μL of water, denaturing at 80°C for 90 sec then incubating for 1 hr at 37°C with 5 μL of 10 X PNK reaction buffer, 1 μL SuperAse-In and 1 μL of T4 polynucleotide kinase. The PNK was inhibited by incubating at 70°C for 10 min, and the sample washed once with 500 μL Wash buffer 2 from the Click-iT kit before resuspending them in 9 μL of 10 mM Tris-HCl pH8. A preadenylylated linker (1 μL of 0.5 μg/μL) (Universal miRNA Cloning Linker, NEB, S1315S) was then ligated after a brief denaturation of 90 sec at 80°C, with T4 truncated RNA ligase 2 (T4 Rnl2(tr), NEB M0242L), for 30 min at 24°C then overnight at 16°C, shaking at 1,000 rpm to avoid the beads from pelleting down. The next day, the samples were washed with 500 μL wash buffer 1 then 500 μL wash buffer 2 and resuspended in 10 μL of 10 mM Tris-HCl pH8. Reverse transcription was performed on the beads with 2 μL of 1.25 μM of reverse transcription primer oJC3453 [94] and Superscript III (kit 18080–400 from Thermofisher scientific) as in the Superscript III kit protocol, incubating at 50°C at 900 rpm for 1h. The cDNA was then eluted from the beads by heating at 90°C for 8min, mixing at 1,200 rpm, and immediately collected from the beads. After isopropanol precipitation, the cDNAs were purified on a 15% denaturing polyacrylamide gel as described above, excising the product above the unextended reverse transcription primer. After extraction from the gel overnight (400 μL of DNA gel extraction buffer: 300 mM NaCl, 10 mM Tris-HCl pH8, 1 mM EDTA) and isopropanol precipitation, the cDNAs were circularized with CircLigase (Epicenter, CL4115K) following the manufacturer’s protocol. A tenth of the circularized cDNA was used for each 20 μL PCR amplification reaction (0.2 μL of Phusion polymerase, 0.2 mM dNTPs and 0.5 μM forward and reverse library primers (different indexing reverse primers to multiplex samples, see S1 Table)). After checking the libraries obtained at 10, 12, 14 cycles (1 min initial denaturation at 98°C, then cycles of 30 sec at 98°C, 30 sec at 65°C and 20 sec at 72°C), 12 cycles was chosen as the best number of cycles to avoid the formation of reannealed partial duplex library products. The rest of the cDNA was used in 12 cycles reactions and separated by electrophoresis (8% polyacrylamide nondenaturing gels, NuPAGE from thermofisher scientific), excised from the gel (avoiding any lower product band derived from unextended reverse transcription primer) and pooled together for each time-point. The libraries were resuspended in 15 μL 10 mM Tris-HCl pH8 and sequenced on an Illumina HiSeq 2500 using single read 50 cycles runs.

### Single-gene transcription shutoff/mRNA decay analysis by northern blot

Prior to decay analysis, HEK293 Tet-Off^®^ cells were plated in DMEM + 10% Tet-free FBS (Tet System Approved FBS; Clontech 631106) + 1% Penicillin/Streptomycin. Plasmid DNA was transfected into HEK293 Tet-Off^®^ cells using the Lipofectamine 2000 reagent (Thermo Fisher Scientific 11668027) protocol; transfection media was removed 24 hours post-transfection. At 48 hours post-transfection, media was replaced with DMEM + Tet-free FBS + 2 ug/mL doxycycline (Sigma-Aldrich Cat# D3072). Cells were harvested using 1 mL Trizol (Thermo Fisher Scientific 15596018) at indicated timepoints. RNA was obtained from mammalian cells using the Trizol RNA isolation protocol with isopropanol precipitation and two 70% ethanol washes.

For steady-state mRNA analyses, cells were plated and transfected as above; cells were harvested using 1 mL Trizol (Thermo Fisher Scientific 15596018) 48 hours post-transfection.

Agarose Northern analyses were performed as described in [28]. Briefly, RNA was loaded onto 1.4% formaldehyde agarose gels and run at 100V for 90 min. Gels were imaged to check for ribosomal RNA quality and quantity before blotting onto Hybond-N+ membrane (GE Amersham RPN303B) overnight by capillary action transfer.

All Firefly luciferase, *MECP2*, *CFTR*, *CRIP1*, *LSM8*, and *SPTSSA* reporter transcripts were detected using a ^32^P-α-CTP (Perkin-Elmer; 3000 Ci/mmol) radiolabeled asymmetric PCR probe directed towards the pJC842 synthetic 3’-UTR (oJC3609/10; see S1 Table). Probe was hybridized overnight at 65°C, then washed twice using 2X SSC/0.1% SDS at 24°C (5 min each) followed by extensive washing (1 hr) in 0.1% SSC/0.1% SDS at 50°C.

U6 snRNA and actin northern probes were created by end-labeling U6 snRNA and actin oligo probes (see S1 Table) with ɣ-^32^P-ATP (Perkin Elmer; specific activity = 6000 Ci/mmol) using T4 Polynucleotide Kinase (New England Biolabs, Cat. M0201S). Probe was hybridized overnight at 42°C, then washed twice in 6X SSC/0.1% SDS at 24°C for 15 min, then at 50°C for 15 min.

Blots were exposed on a storage phosphor screen for 15 min (U6 snRNA) or overnight (Firefly, *MECP2*, *CFTR*, *CRIP1*, *LSM8*, and *SPTSSA* reporters). Stored signal was read using the Typhoon 9400 Variable Mode Imager (Amersham Biosciences). Quantitation of phosphorimager signal was performed using ImageQuant software (Molecular Dynamics; version 5.2).

Polyacrylamide Northern analysis of *SPTSSA* reporter mRNA was performed using 6% polyacrylamide/urea denaturing gels. Samples were run at 400V for 16 hr in 1X TBE and transferred at 50V in 0.5X TBE for 3 hours at 4°C. Hybridization with radiolabeled asymmetric PCR probe, washing, and detection proceeded as described above.

### tRNA sequencing in HeLa cells

Total RNA was isolated from HeLa cells using TRIzol. 1ug of total RNA was ligated to annealed adapters under conditions as in [47]. cDNA was synthesized using SuperScript IV Reverse Transcriptase (ThermoFisher 18090010) using manufacturer’s recommended conditions and gel purified with denaturing polyacrylamide gel electorphoresis. Gel purified product was then ligated using CircLigase (Lucigen CL4111K) using manufacturer’s recommended conditions and libraries were amplified using Q5 DNA polymerase (New England Biolabs M0491) for 7–8 cycles then purified from a 2% agarose gel. Libraries were sequenced as single-end reads on an Illumina NextSeq 550.

### Free amino acid analysis

HeLa cells were plated in DMEM + 10% BCS + 4 mM L-Glutamine and grown to approximately 80% confluency. Cells were transferred to ice and washed briefly in ice-cold 1X PBS. Cells were scraped into 1 mL ice-cold 10% trichloroacetic acid (TCA; Fisher Scientific A322-100) and homogenized by passing through a 22-gauge needle ~10 times. Lysates were incubated on ice 1 hr to allow protein precipitation before centrifuging at 5,000 x *g* for 15 min at 4°C. Supernatant containing free amino acids was transferred to a new 1.5 mL microcentrifuge tube and stored at -80°C prior to analysis.

Free intracellular amino acid analysis was performed by the Proteomics and Peptide Synthesis Core of the University of Michigan Medical School Biomedical Research Core Facilities. 20 uL cell lysate was derivatized with *o*-phthalaldehyde (OPA) and 9-fluoromethyl-chloroformate (FMOC) before separation and quantitation of amino acids by reverse-phase high performance liquid chromatography (HPLC) using the Hewlett Packard AminoQuant method on an Agilent 1260 liquid chromatograph. System control and data analysis was performed using Agilent Chemstation software.

## Statistical analysis

### HeLa mRNA half-life data analysis

Raw and processed BRIC-Seq half-life data for wild type HeLa cells was obtained from the Gene Expression Omnibus under accession GSE102113. See [56] for details on data processing, including alignment, quantification, filtering, and half-life calculations.

### CHO global mRNA half-life data analysis

Adapter sequence was clipped from raw reads (CTGTAGGCACCATCAAT) with fastx_clipper, and reads smaller than 18 nucleotides were discarded. The reads were then aligned to the *Cricetulus griseus* genome (GCF_000223135.1_CriGri_1.0) using hisat2 v2.1.0, converted into bam format and indexed using samtools v.1.7–2. Transcript FPKM values were calculated with Stringtie v.1.3.5 and Ballgown [96] using default parameters and a gtf file of the CriGri_1 genome downloaded from RefSeq as annotation file. This file was used to create a merged transcripts annotation used in Stringtie to re-estimate the transcripts abundances that were given as input to Ballgown. The raw FPKM numbers at each time-point were normalized to the relative number of reads aligning to the spike-ins (average of reads aligning to Luc and LYSa normalized to the number of total reads) to adjust for the amplification resulting from a smaller pool of transcripts at the later time-points. Transcripts with less than 1 FPKM at time-point 0 and without a corresponding protein-coding sequence were filtered out. Half-lives were calculated as in [28]. Briefly, spike-in-normalized FPKM values for each timepoint were further normalized to the 0 hr value. Half-lives were estimated by fitting a least absolute deviations regression model. The decay equation model took into account the estimated doubling time of the cells (15 hr) to correct for the dilution due to cell growth. Resultant half-lives were filtered to exclude genes which had an estimated half-life longer than 18 hr and for which the average absolute residual was more than 20.

### Determination of relative tRNA levels by codon using tRNA sequencing

Following CCA trimming, tRNA sequencing reads were aligned to high confidence tRNA genes from GtRNAdb (hg38) with Bowtie2. Read counting over tRNA genes, reads per million (RPM), and subsequent tAI calculations were performed in R using the Rsubread package [97]. Selenocysteine (Sec) and initiator methionine (iMet) reads were excluded from downstream analysis. tRNA-Seq based HEK293T and HeLa tAIs are listed in S2 Table.

### Codon and amino acid stabilization coefficient calculations

CSC and AASC calculations were performed for all organisms using half-life datasets and FASTA-formatted protein-coding gene sequences (citations and accession numbers detailed in S3 Table). All coding sequences were obtained from ensembl.org except *Schizosaccharomyces pombe* (obtained from pombase.org) and *Cricetulus griseus* (obtained from RefSeq genomes; https://www.ncbi.nlm.nih.gov/genome). For transcript identifiers with more than one coding sequence, the longest sequence was selected. Coding sequences were further filtered to obtain only sequences starting with “ATG” for downstream analysis. Half-life datasets were filtered to obtain half-lives > 0. Phylogenetic tree diagram showing evolutionary relationships between different species considered in this study was prepared using the Interactive Tree of Life (iTOL) online tool [98].

To calculate codon stability coefficients (CSCs), transcript-specific codon frequencies were obtained using the oligonucleotideFrequency function in the Biostrings R package (version 2.50.1; [99]) with width = 3, step = 3, as.prob = TRUE to normalize to transcript length. CSCs were obtained by calculating the Pearson correlation coefficient between transcript half-life and codon frequency for all 61 non-stop codons using the cor.test function in the R stats package (version 3.5.1; [100]) with method = “pearson”.

To calculate amino acid stabilization coefficients (AASCs), coding sequences were translated into amino acid sequences using the translate function in the Biostrings R package with genetic.code = GENETIC_CODE and if.fuzzy.codon = “X”. Transcript-specific amino acid frequencies were obtained using the alphabetFrequency function with as.prob = TRUE to normalize to transcript length. AASCs were obtained by calculating the Pearson correlation coefficient between transcript half-life and amino acid frequency for all 20 canonical amino acids using the cor.test function in the R stats package with method = “pearson”.

### Half-life comparisons for homopolymeric amino acid repeat-containing genes

A list of protein-coding genes containing homopolymeric amino acid repeats was obtained from [57]. Protein IDs were converted to HGNC gene symbol before merging with HeLa half-life data. Gene list was filtered for homopolymeric repeat stretches of highly stabilizing (V, I, Y, A) and destabilizing (S, H, Q, C) amino acids. For genes containing more than one repeat stretch for any given amino acid, the longest stretch was selected using the top_n function in R.

### Gene groups analysis and transcript-level CSC and AASC calculations

Gene symbols for various protein families or related pathways were retrieved from the Gene Ontology Consortium (geneontology.org/; TCA cycle: GO:0006099; ATP synthesis: GO:0006754; Glycolysis: GO:0006096; Splicing proteins: GO:0000398; Cell cyle proteins: GO:0007049), the InterPro Protein Sequence Analysis and Classification database (http://www.ebi.ac.uk/interpro/; Protein kinase family: IPR017892; KH Domain protein family: IPR004088), and the HUGO Gene Nomenclature Committee (http://genenames.org; Zinc finger proteins Group ID: 26; Serine/Arginine-rich proteins Group ID: 737; Cytoplasmic ribosomal proteins Group IDs: 728 + 729; Mitochondrial ribosomal proteins Group ID: 646).

For gene groups analysis, additional cytoplasmic ribosomal protein transcripts (HGNC Group IDs 728 + 729) were identified which otherwise passed filtering (i.e. adequate read depth across the entire timecourse for both HeLa BRIC-Seq replicates), yet failed half-life estimation by linear modeling due to high stability; these transcripts were manually assigned the maximum half-life (24 hrs) and added to existing half-life data. Transcript-level average CSC and AASCs were calculated as follows:

$$Transcript\;CSC=\sum ({Codon.freq}_{AAA}*{CSC}_{AAA})+\cdots +({Codon.freq}_{TTT}*{CSC}_{TTT})$$

$$Transcript\;AASC=\sum ({AA.freq}_{A}*{AASC}_{A})+\cdots +({AA.freq}_{W}*{AASC}_{W})$$

for all 61 non-stop codons and all 20 canonical amino acids. All gene group designations, half-life data, and transcript-level CSC and AASC values are detailed in S5 Table.

### Other statistical analyses

Number of replicates, statistical tests used, and appropriate correlations and/or *p*-values are specified in the figures and figure legends. All tests were performed at a 95% confidence level.

## Supporting information

S1 Raw images (PDF)

S1 Fig Optimal codon content modulates mRNA stability in human cells (related to Fig 1). (A) Two by two table detailing proportions of optimal vs non-optimal codons (as defined by HEK293T tRNA sequencing) with G or C at the at the 3’-nucleotide position (GC3) versus A or T (AT3). P-value and confidence intervals indicate results of Fisher’s exact test for difference in proportion of GC3 versus AT3 codons designated as optimal. (B) Plot of frequency of codons with G or C at the 3’-nucleotide position (GC3) within variable optimality Firefly reporter ORF sequences. (C) Northern blot analysis of U6 snRNA as a loading control for mRNA decay analysis of Firefly luciferase variable codon optimality reporters in Fig 1C. Timepoints represent time elapsed after shutoff of transcription with doxycycline. (D) Northern blot analysis of U6 snRNA as a loading control for mRNA decay analysis of *MECP2* variable codon optimality reporters in Fig 1D. Timepoints represent time elapsed after shutoff of transcription with doxycycline. (E) Northern blot analysis of actin mRNA as a loading control for mRNA decay analysis of *CFTR* ΔF508 variable codon optimality reporters in Fig 1E. Timepoints represent time elapsed after shutoff of transcription with doxycycline. (F) Scatterplot comparing codon stability coefficients (CSC) calculated from HeLa endogenous mRNA half-life datasets used in this study and in a parallel study by Wu and colleagues [41]. *R* = 0.6, *p* = 3.0 x 10^−7^ (Pearson correlation test). (G) Scatterplot comparing codon stability coefficients (CSC) between HeLa and CHO datasets. Dark blue line represents linear regression trendline. Codons indicated in magenta are significantly stabilizing or destabilizing in both datasets; *R* = 0.88, *p* < 2.2 x 10^−16^ (Pearson correlation test). See also S1 Table for northern probe sequences and reporter ORF sequences and S2 Table for tAI and CSC values.
(PDF)

S2 Fig mRNAs containing destabilizing amino acid stretches show accelerated deadenylation (related to Fig 3). (A) Scatterplot comparing amino acid stabilization coefficients (AASC) between HeLa and CHO datasets. Dark blue line represents linear regression trendline. Amino acids indicated in magenta are significantly stabilizing or destabilizing in both datasets; *R* = 0.91, *p* = 3.1 x 10^−8^ (Pearson correlation test). (B) Northern blot analysis of U6 snRNA as a loading control for transcription shutoff/mRNA decay analysis of *CRIP1*, *LSM8*, and *SPTSSA* amino acid stretch reporters in Fig 2G. (C) (top) High resolution polyacrylamide northern blot analysis of *SPTSSA* amino acid stretch reporters. A_F_ arrow indicates transcripts with full-length polyA tails; A_0_ arrow indicates fully deadenylated transcript. Timepoints represent time elapsed after shutoff of transcription with doxycycline. (bottom) Barplots showing distribution of northern blot signal over time ranging from full-length polyA tail (A_F_) to bulk polyA tail removal (A_0_) and separated into four bins of equal length. Lineplot of relative full-length polyA tail remaining over time is shown at the far right.
(PDF)

S3 Fig Encoded amino acids are associated with mRNA stability to a lesser extent in *S*. *cerevisiae* than HeLa (related to Fig 3). (A) Plot of amino acid stabilization coefficients (AASC) for *S*. *cerevisiae* mRNAs (n = 3,890). Blue represents significantly stabilizing amino acids; red represents significantly destabilizing amino acids (Pearson correlation test; *p* < 0.01). Dotted line indicates genome-wide significance level (*p* < 5 x 10^−8^; note that no destabilizing amino acids reached this significance level). Error bars indicate confidence interval about Pearson *R* estimate. (B) Boxplots of *S*. *cerevisiae* mRNA half-life distributions binned by alanine frequency. Number of transcripts in bin is indicated above each boxplot. Overall difference in means *p* < 2.2 x 10^−16^ (Kruskal-Wallis test). (C) Boxplots of *S*. *cerevisiae* mRNA half-life distributions binned by serine frequency. Number of transcripts in bin is indicated above each boxplot. Overall difference in means *p* = 8.5 x 10^−12^ (Kruskal-Wallis test). See also S2 Table for AASC values and statistical data and S3 Table for half-life data source.
(PDF)

S4 Fig The association between HeLa CSC and tRNA levels is weakened upon frameshifting (related to Fig 4). (A) Two by two table detailing proportions of optimal vs non-optimal codons (as defined by HeLa tRNA sequencing) with G or C at the at the 3’-nucleotide position (GC3) versus A or T (AT3). P-value and confidence intervals indicate results of Fisher’s exact test for difference in proportion of GC3 versus AT3 codons designated as optimal. (B) Boxplots of HeLa mRNA distributions binned by total optimal codon frequency (sum of codon frequencies for optimal codons, as defined in Fig 4A). Overall difference in means *p* < 2.2 x 10^−16^ (Kruskal-Wallis test). (C) Scatterplots of HeLa tAI versus CSCs calculated from ORF sequences frame-shifted by one nucleotide (F+1; top) and ORF sequences frame-shifted by two nucleotides (F+2; bottom) for all 61 codons. Spearman rho (ρ) and P-values indicate results of Spearman correlation test. (D) Scatterplots of HeLa tAI versus CSCs calculated from ORF sequences frame-shifted by one nucleotide (F+1; top) and ORF sequences frame-shifted by two nucleotides (F+2; bottom) for the 42 codons encoding amino acids with moderate effects on mRNA stability (-0.10 < AASC < 0.10, *p* > 10^−30^). Spearman rho (ρ) and P-values indicate results of Spearman correlation test. (E) Scatterplots of HeLa intracellular amino acid levels versus CSCs calculated from ORF sequences frame-shifted by one nucleotide (F+1; top) and ORF sequences frame-shifted by two nucleotides (F+2; bottom) for the 19 codons encoding the 6 amino acids with extreme effects on mRNA stability (AASC > 0.10 or < -0.10; *p* < 10^−30^). Spearman rho (ρ) and P-values indicate results of Spearman correlation test.
(PDF)

S5 Fig Overall codon and amino acid composition are more predictive of endogenous HeLa half-lives than GC3 codon frequency or GC content. (A) Histograms showing distribution of untransformed HeLa endogenous mRNA half-lives (left) and HeLa half-lives after reciprocal transformation (-1/half-life; right). Red curve indicates a theoretical normal distribution with identical mean and standard deviation for each dataset. (B) Density plots showing distribution of values for transcript-average CSC, transcript-average AASC, GC3 codon frequency, and GC content. (C) Correlation matrix showing Pearson correlation estimates for each combination of the five indicated variables. -1/Half-Life = transformed HeLa mRNA half-lives. GC3 Codons = combined frequency of codons with G or C at the 3’-position. (D) Boxplots comparing HeLa half-life distributions for increasing transcript-average CSC (top) and GC3 codon frequency (bottom). P-values indicate overall difference in means across all bins (Kruskal-Wallis test).
(PDF)

S6 Fig Codon and amino acid-based effects on stability vary across different species (related to Fig 6). (A-B) Plots of codon stability coefficients (CSC) grouped by encoded amino acid for (A) *Danio rerio* (zebrafish) and (B) *Drosophila melanogaster*. Magenta indicates amino acids encoded exclusively by stabilizing (CSC > 0) or destabilizing (CSC < 0) synonymous codons. See also Fig 6C for phylogenetic tree diagram, S2 Table for species-specific CSC values, and S3 Table for half-life dataset sources.
(PDF)

S1 Table Plasmids and oligonucleotide sequences. List of northern blot probe sequences, plasmid IDs, and reporter open reading frame insert sequences used in this study.
(XLSX)

S2 Table tRNA adaptive indices (tAI), free amino acid levels, codon stability coefficients (CSC), and amino acid stabilization coefficients (AASC). Consolidated list of tRNA adaptive index (tAI) values for HEK293T and HeLa cells, relative free amino acid concentrations for HeLa cells, codon stability coefficient (CSC) values for all species, and amino acid stabilization coefficient (AASC) values for HeLa, CHO, and *S*. *cerevisiae*.
(XLSX)

S3 Table List of species-specific half-life datasets and FASTA coding sequence sources. List of sources for half-life datasets and FASTA coding sequence lists used for CSC and AASC calculations for all species listed in this study.
(XLSX)

S4 Table Homopolymeric amino acid stretch half-life data. List of genes containing homopolymeric amino acid stretches (adapted from [57]) and associated amino acid repeat identity, length, and HeLa half-life.
(XLSX)

S5 Table Gene groups analysis half-life data. List of gene group designations, half-life data, and transcript average CSC and AASC scores for HeLa (S5A) and *S*. *cerevisiae* (S5B).
(XLSX)
