# Supplementary material for: Codon and amino acid content are associated with mRNA stability in mammalian cells
Source: PLoS One. 2020 Feb 13;15(2):e0228730. doi: 10.1371/journal.pone.0228730 (PMC7018022; doi:10.1371/journal.pone.0228730)
Supplement: S1 Raw images — (PDF) [file pone.0228730.s001.pdf]

S1_raw_images Forrest et al.

Fig 1B: Firefly luciferase variable optimality steady-state mRNA + U6 snRNA loading control

Radiographic image detected after exposure on phosphor storage screen
Instrument: Typhoon 9400 variable mode imager (storage phosphor setting)

Probe: ^{32}P-CTP reporter synthetic 3'-UTR (Firefly luciferase)
Exposure time: ~16 hr (overnight)

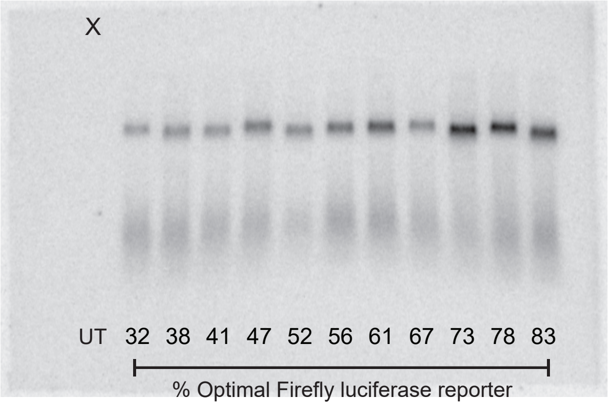

UT = untransfected cells
X = lane not included in figure

Probe: ^{32}P-ATP U6 snRNA
Exposure time: 15 min

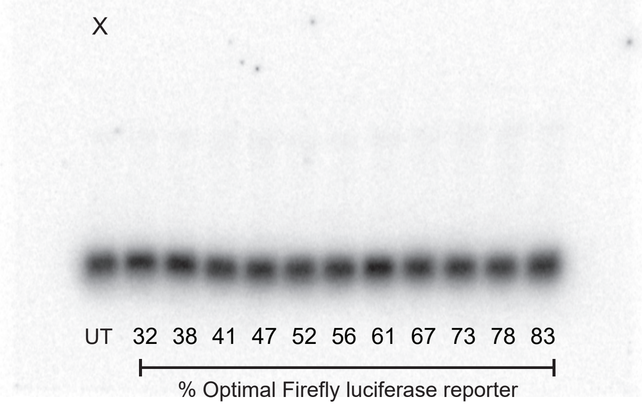

UT = untransfected cells
X = lane not included in figure

Fig 1C: Firefly luciferase variable optimality single-gene mRNA decay analysis

Radiographic image detected after overnight exposure (~16 hrs) on storage phosphor screen
Instrument: Typhoon 9400 variable mode imager (storage phosphor setting)
Probe: ^{32}P-CTP reporter synthetic 3'-UTR (Firefly luciferase)

Firefly luciferase
32% optimal

Firefly luciferase
56% optimal

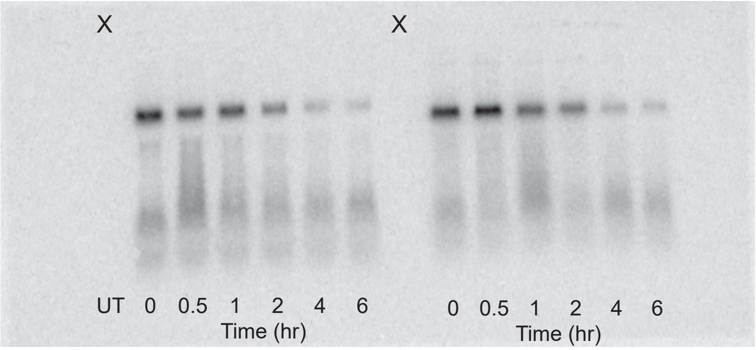

UT = untransfected cells
X = lane not shown in figure

(Data not shown)

Firefly luciferase
83% optimal

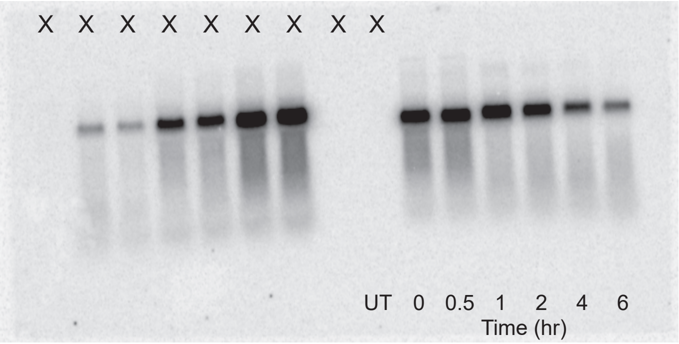

UT = untransfected cells
X = lane not shown in figure

S1_raw_images Forrest et al.

Fig 1D: *MECP2* variable optimality single-gene mRNA decay analysis

Radiographic image detected after overnight exposure (~16 hrs) on storage phosphor screen
Instrument: Typhoon 9400 variable mode imager (storage phosphor setting)
Probe: ^{32}P-CTP reporter synthetic 3'-UTR (*MECP2*)

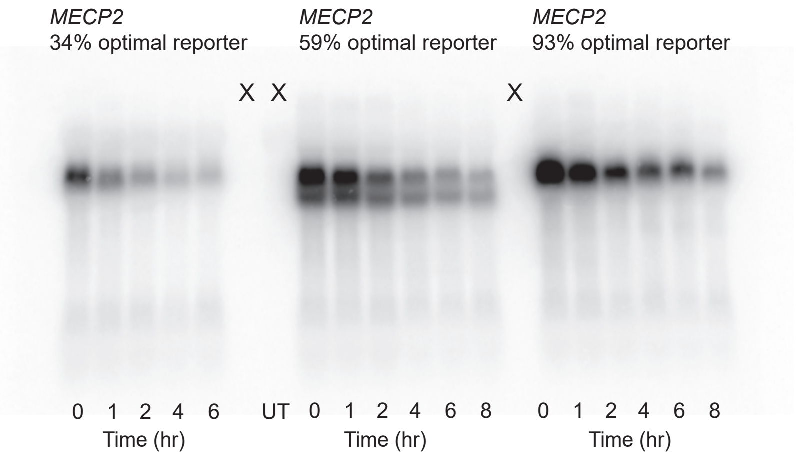

UT = untransfected cells
X = lane not included in figure

Fig 1E: *CFTR* variable optimality single-gene mRNA decay analysis

Radiographic image detected after overnight exposure (~16 hrs) on storage phosphor screen
Instrument: Typhoon 9400 variable mode imager (storage phosphor setting)
Probe: ^{32}P-CTP reporter synthetic 3'-UTR (*CFTR*)

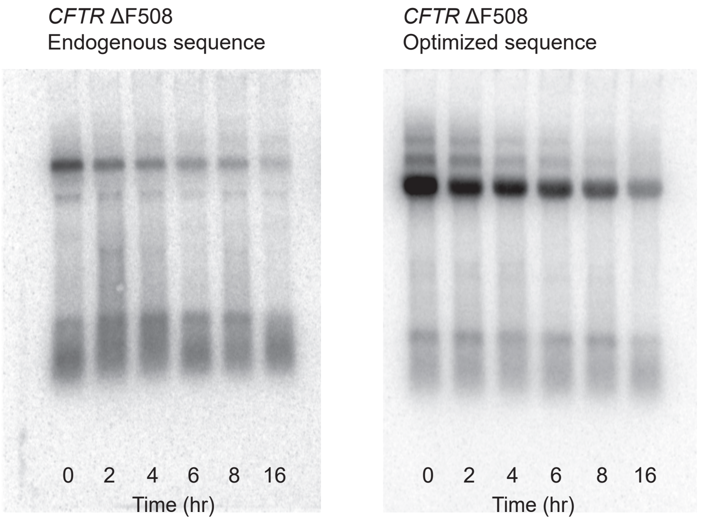

S1_raw_images Forrest et al.

Fig 3G: *CRIP1* amino acid stretch single-gene mRNA decay analysis

Radiographic image detected after overnight exposure (~16 hrs) on storage phosphor screen
Instrument: Typhoon 9400 variable mode imager (storage phosphor setting)
Probe: ^{32}P-CTP reporter synthetic 3'-UTR (*CRIP1*)

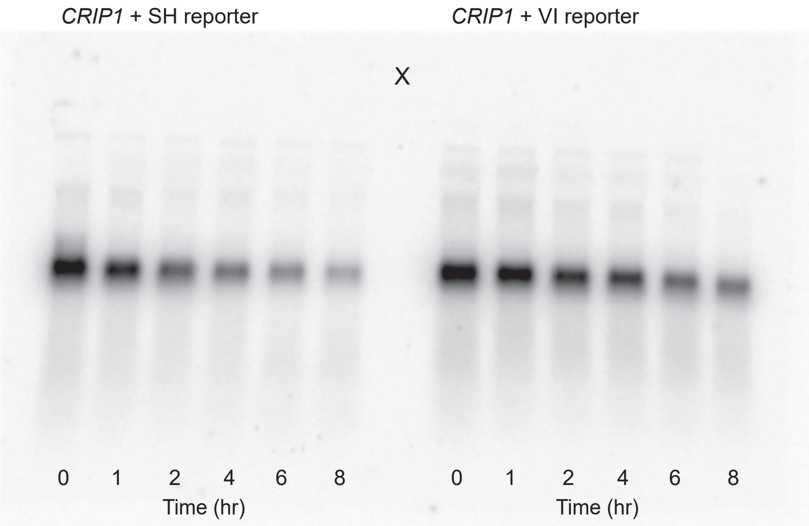

X = lane not shown in figure

Fig 3G: *LSM8* amino acid stretch single-gene mRNA decay analysis

Radiographic image detected after overnight exposure (~16 hrs) on storage phosphor screen
Instrument: Typhoon 9400 variable mode imager (storage phosphor setting)
Probe: ^{32}P-CTP reporter synthetic 3'-UTR (*LSM8*)

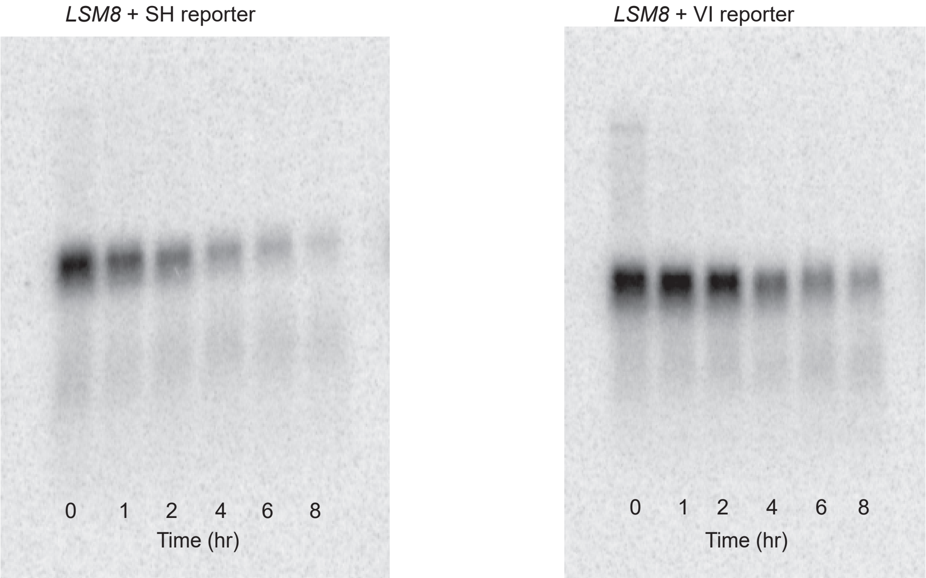

S1_raw_images Forrest et al.

Fig 3G: *SPTSSA* amino acid stretch single-gene mRNA decay analysis

Radiographic image detected after overnight exposure (~16 hrs) on storage phosphor screen
Instrument: Typhoon 9400 variable mode imager (storage phosphor setting)
Probe: ^{32}P-CTP reporter synthetic 3'-UTR (*SPTSSA*)

SPTSSA + SH reporter   *SPTSSA* + VI reporter

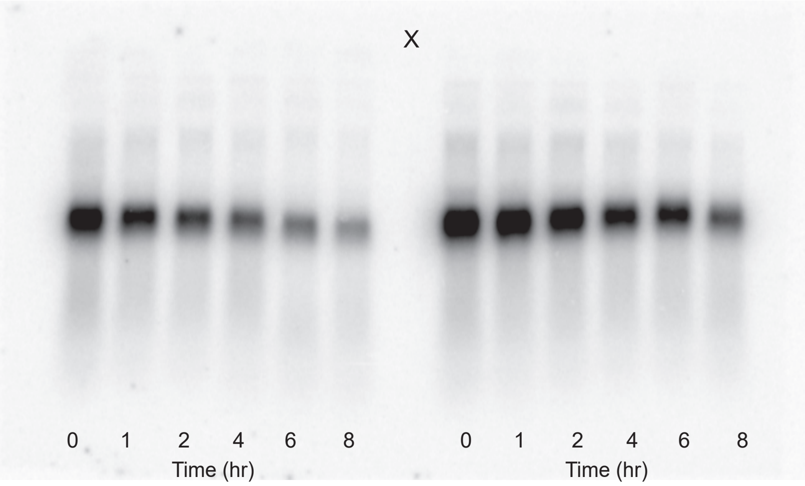

X = lane not shown in figure

S1C Fig: U6 snRNA loading control for Firefly luciferase variable optimality mRNA decay analysis

Radiographic image detected after 15 min exposure on phosphor storage screen
Instrument: Typhoon 9400 variable mode imager (storage phosphor setting)
Probe: ^{32}P-ATP U6 snRNA

Firefly luciferase
32% optimal reporter

Firefly luciferase
56% optimal reporter

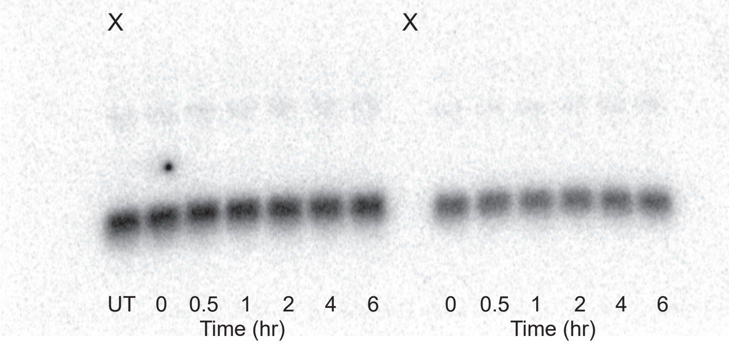

UT = untransfected cells
X = lane not included in figure

(Data not shown)

Firefly luciferase
83% optimal reporter

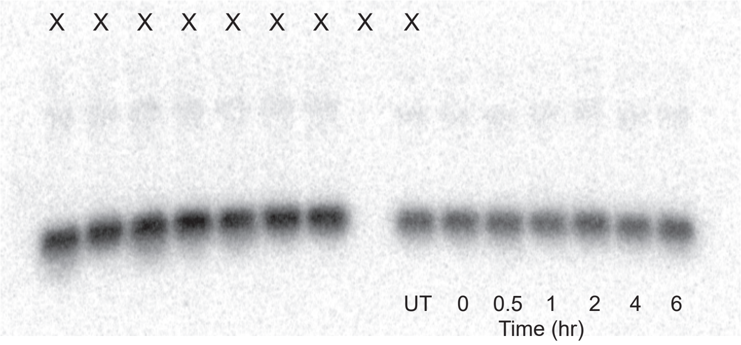

UT = untransfected cells
X = lane not included in figure

S1_raw_images Forrest et al.

S1D Fig: U6 snRNA loading control for *MECP2* variable optimality mRNA decay analysis

Radiographic image detected after 15 min exposure on phosphor storage screen
Instrument: Typhoon 9400 variable mode imager (storage phosphor setting)
Probe: ^{32}P-ATP U6 snRNA

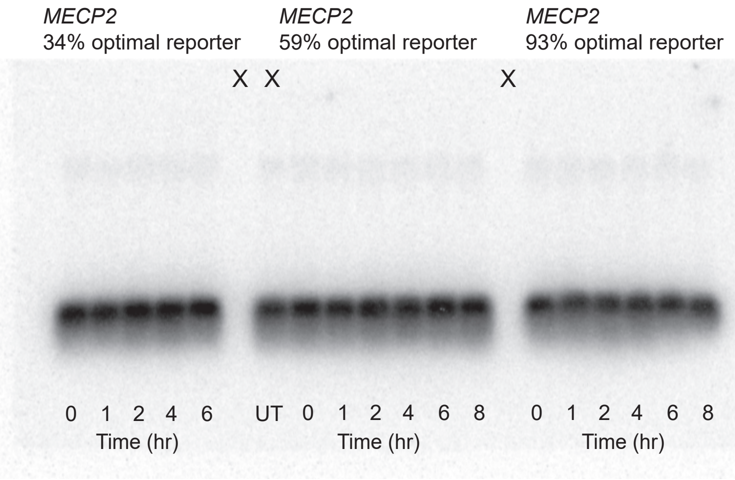

UT = untransfected cells
X = lane not included in figure

S1E Fig: actin mRNA loading control for *CFTR* variable optimality mRNA decay analysis

Radiographic image detected after 15 min exposure on phosphor storage screen
Instrument: Typhoon 9400 variable mode imager (storage phosphor setting)
Probe: ^{32}P-ATP actin mRNA

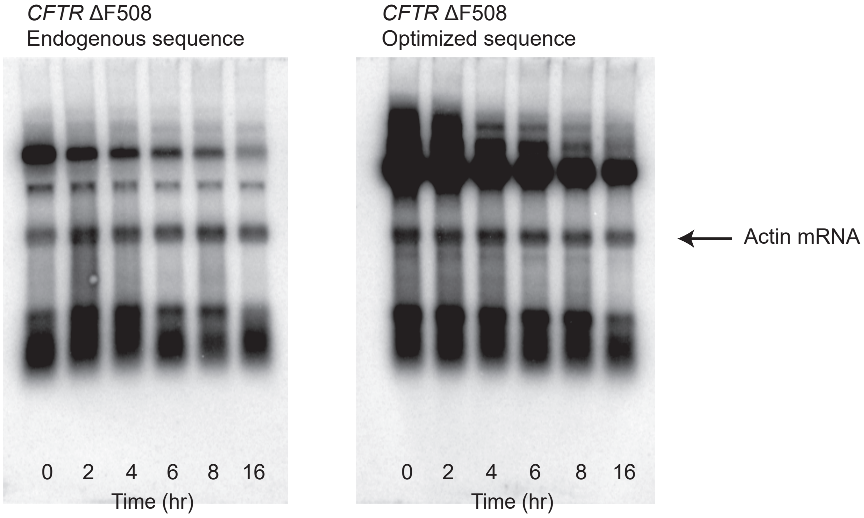

S1_raw_images Forrest et al.

S2B Fig: U6 snRNA loading control for *CRIP1* amino acid stretch mRNA decay analysis

Radiographic image detected after 15 min exposure on phosphor storage screen
Instrument: Typhoon 9400 variable mode imager (storage phosphor setting)
Probe: ^{32}P-ATP U6 snRNA

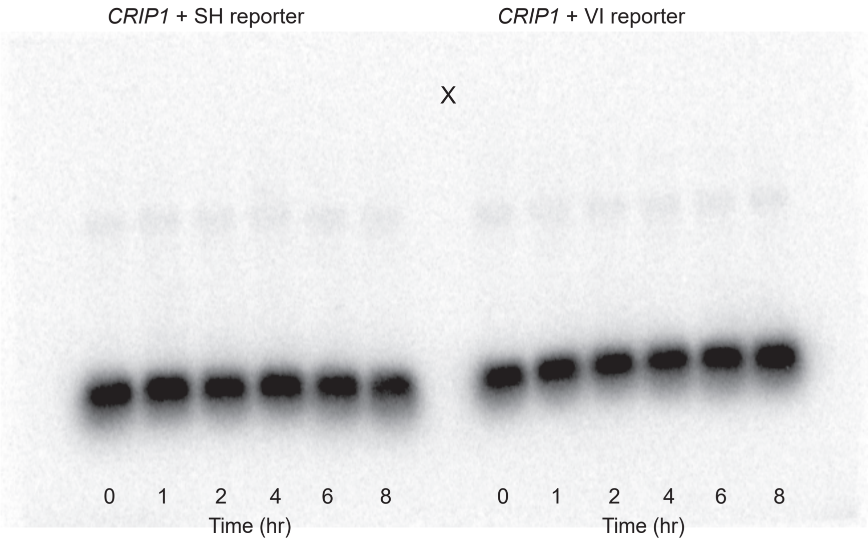

X = lane not shown in figure

S2B Fig: U6 snRNA loading control for *LSM8* amino acid stretch mRNA decay analysis

Radiographic image detected after 15 min exposure on phosphor storage screen
Instrument: Typhoon 9400 variable mode imager (storage phosphor setting)
Probe: ^{32}P-ATP U6 snRNA

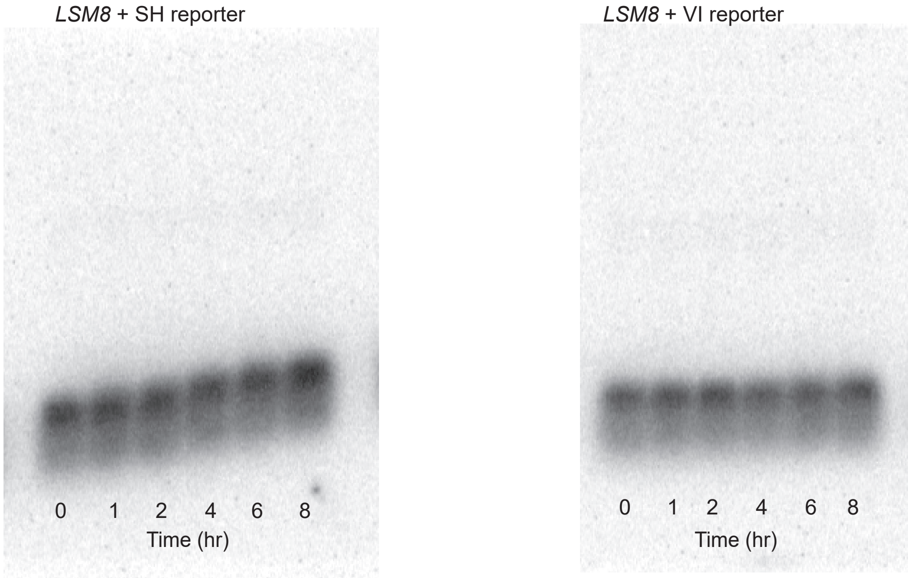

S2B Fig: U6 snRNA loading control for *SPTSSA* amino acid stretch mRNA decay analysis

Radiographic image detected after 15 min exposure on phosphor storage screen
Instrument: Typhoon 9400 variable mode imager (storage phosphor setting)
Probe: ^{32}P-ATP U6 snRNA

SPTSSA + SH reporter *SPTSSA* + VI reporter

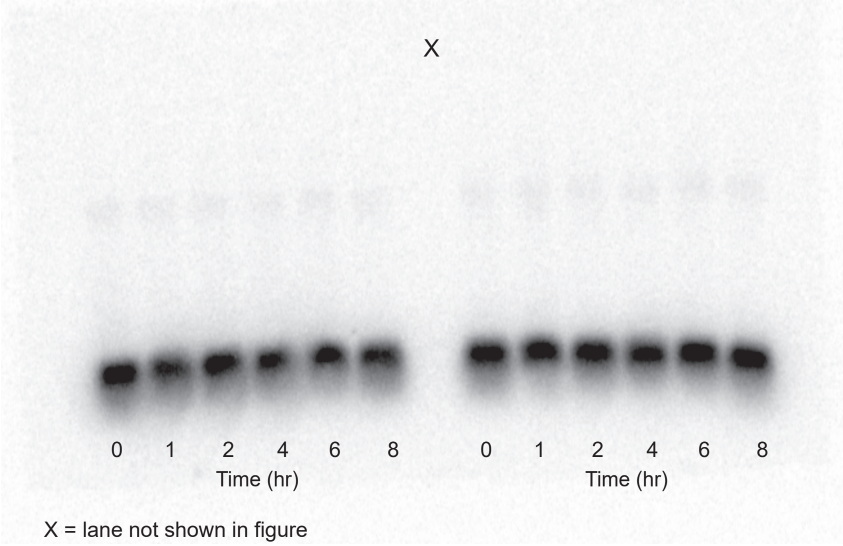

S2C Fig: *SPTSSA* amino acid stretch polyA tail length analysis

Radiographic image detected after 2 day exposure on storage phosphor screen
Instrument: Typhoon 9400 variable mode imager (storage phosphor setting)
Probe: ^{32}P-CTP reporter synthetic 3'-UTR (*SPTSSA*)

SPTSSA + SH reporter

SPTSSA + VI reporter

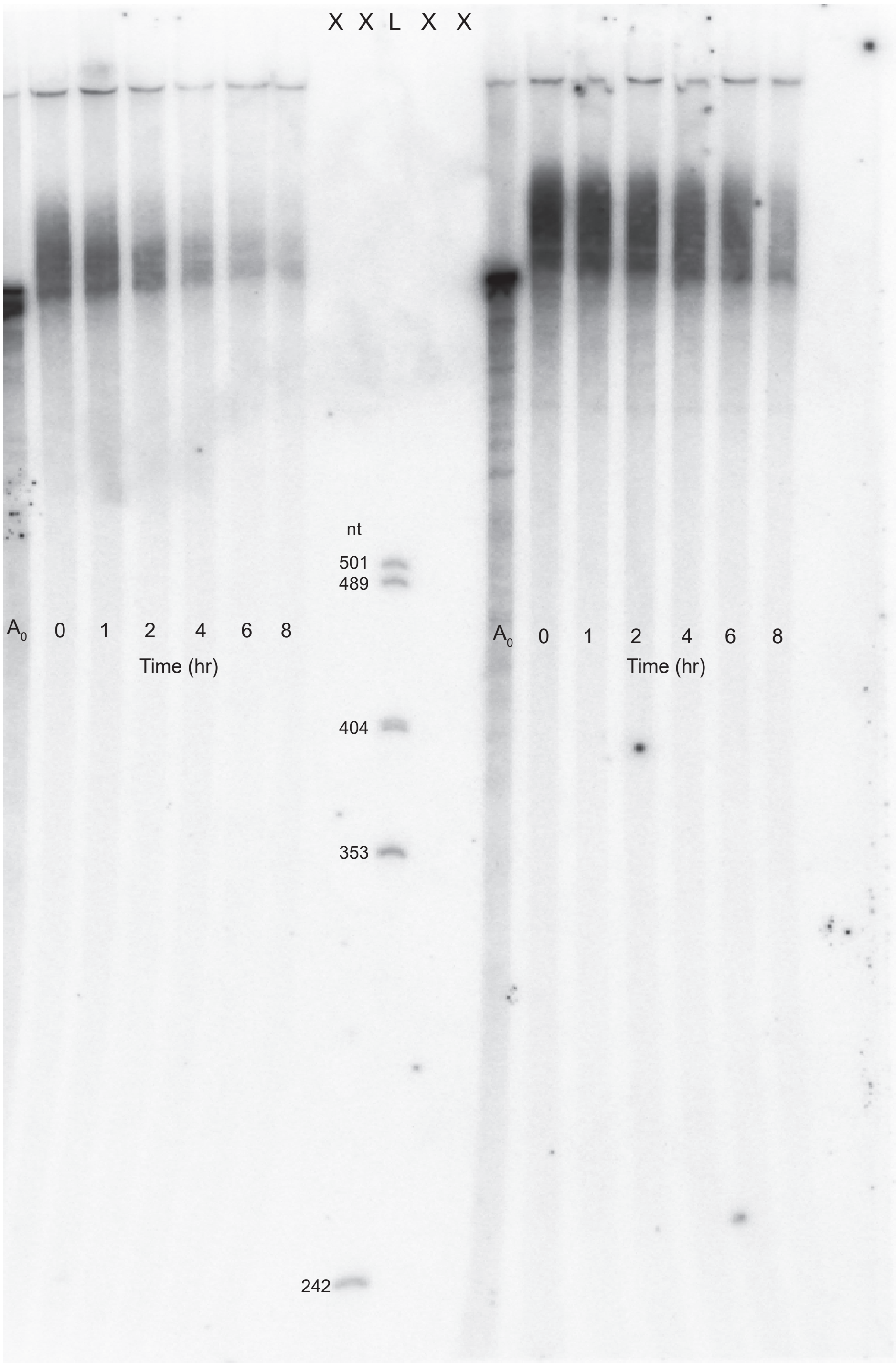

X = lane not shown in figure
L = pUC18 digest ladder (end-labeled with ^{32}P-ATP)
A_0 = deadenylated control
