# Supplementary figures and images for: Codon and amino acid content are associated with mRNA stability in mammalian cells
Source: PLoS One. 2020 Feb 13;15(2):e0228730. doi: 10.1371/journal.pone.0228730 (PMC7018022; doi:10.1371/journal.pone.0228730)

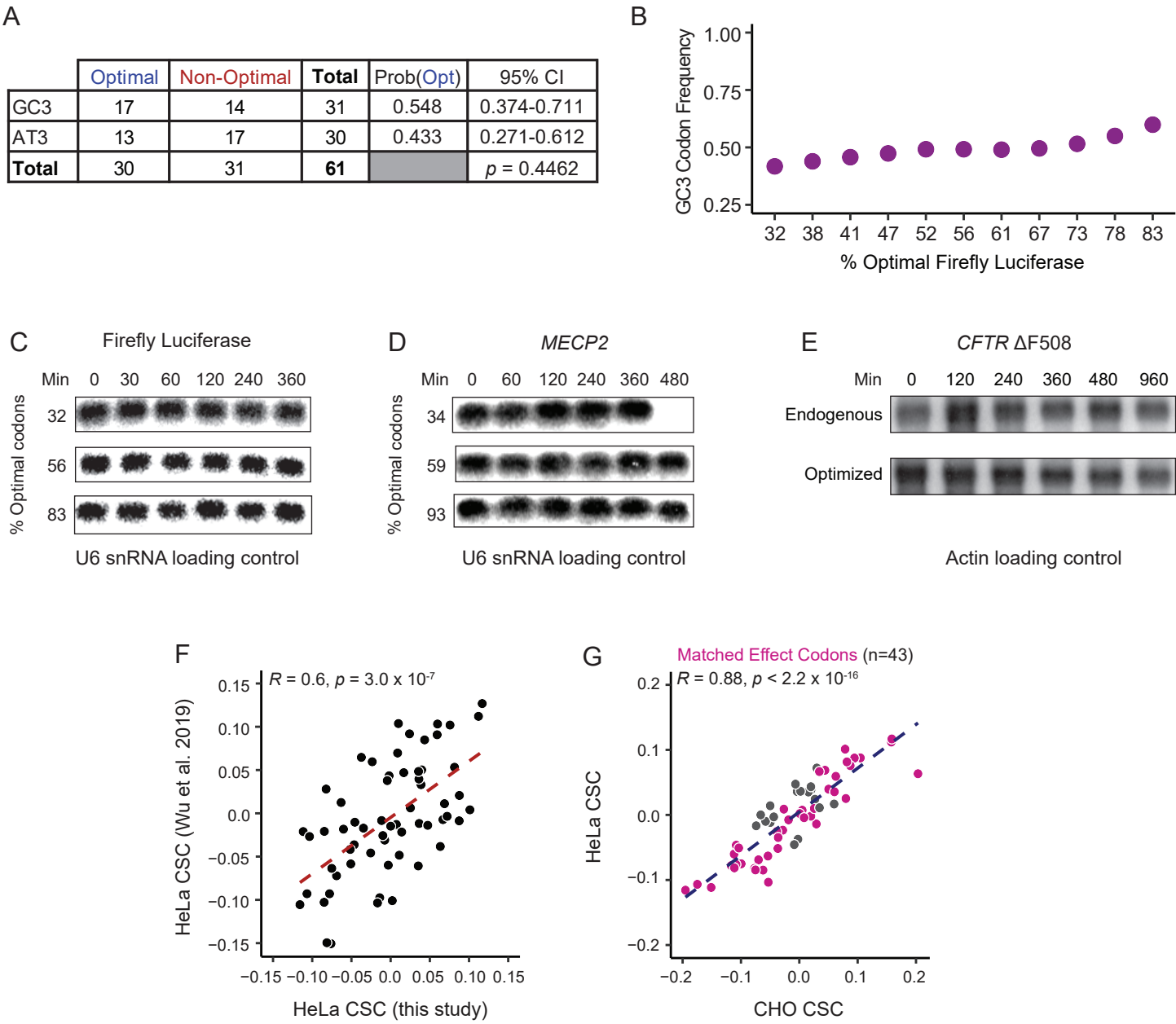

Supplement: S1 Fig — (A) Two by two table detailing proportions of optimal vs non-optimal codons (as defined by HEK293T tRNA sequencing) with G or C at the at the 3’-nucleotide position (GC3) versus A or T (AT3). P-value and confidence intervals indicate results of Fisher’s exact test for difference in proportion of GC3 versus AT3 codons designated as optimal. (B) Plot of frequency of codons with G or C at the 3’-nucleotide position (GC3) within variable optimality Firefly reporter ORF sequences. (C) Northern blot analysis of U6 snRNA as a loading control for mRNA decay analysis of Firefly luciferase variable codon optimality reporters in Fig 1C. Timepoints represent time elapsed after shutoff of transcription with doxycycline. (D) Northern blot analysis of U6 snRNA as a loading control for mRNA decay analysis of MECP2 variable codon optimality reporters in Fig 1D. Timepoints represent time elapsed after shutoff of transcription with doxycycline. (E) Northern blot analysis of actin mRNA as a loading control for mRNA decay analysis of CFTR ΔF508 variable codon optimality reporters in Fig 1E. Timepoints represent time elapsed after shutoff of transcription with doxycycline. (F) Scatterplot comparing codon stability coefficients (CSC) calculated from HeLa endogenous mRNA half-life datasets used in this study and in a parallel study by Wu and colleagues [41]. R = 0.6, p = 3.0 x 10^−7^ (Pearson correlation test). (G) Scatterplot comparing codon stability coefficients (CSC) between HeLa and CHO datasets. Dark blue line represents linear regression trendline. Codons indicated in magenta are significantly stabilizing or destabilizing in both datasets; R = 0.88, p < 2.2 x 10^−16^ (Pearson correlation test). See also S1 Table for northern probe sequences and reporter ORF sequences and S2 Table for tAI and CSC values. (PDF) [file pone.0228730.s002.pdf]

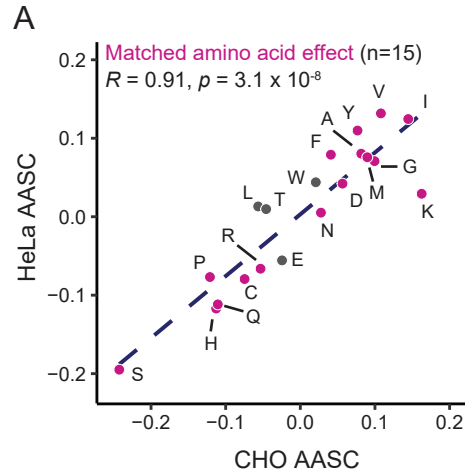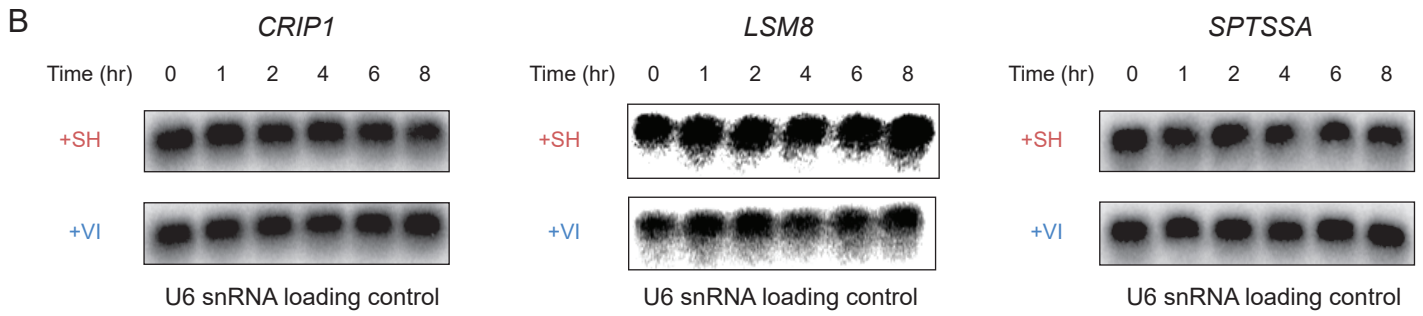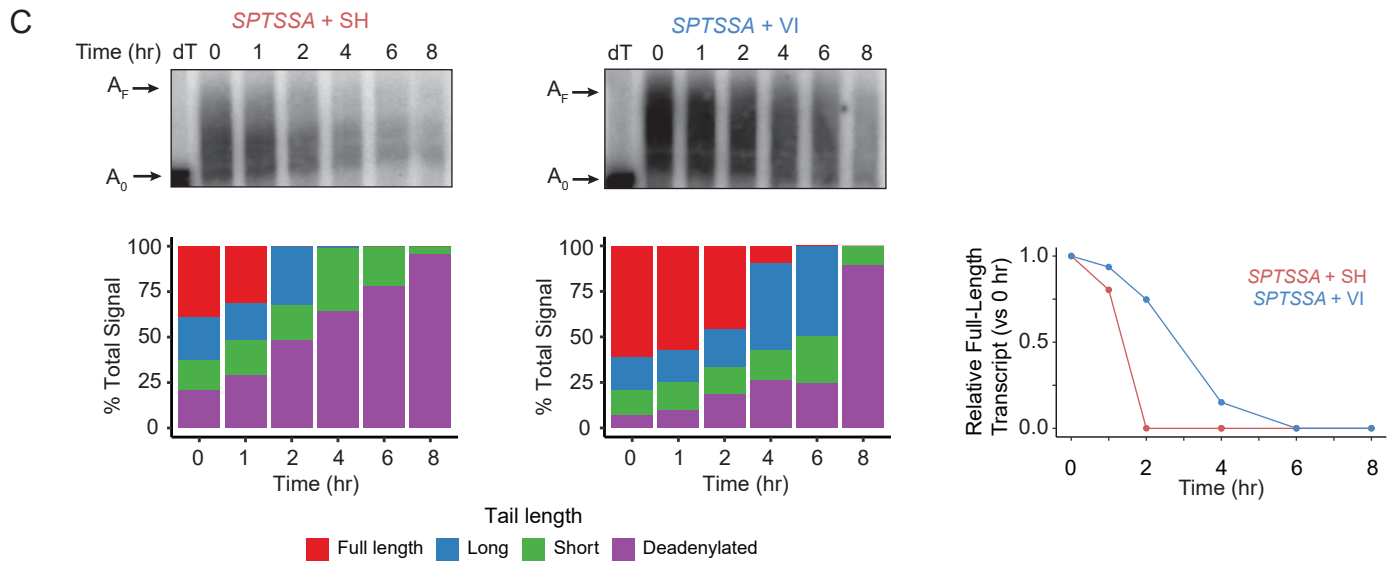

Supplement: S2 Fig — (A) Scatterplot comparing amino acid stabilization coefficients (AASC) between HeLa and CHO datasets. Dark blue line represents linear regression trendline. Amino acids indicated in magenta are significantly stabilizing or destabilizing in both datasets; R = 0.91, p = 3.1 x 10^−8^ (Pearson correlation test). (B) Northern blot analysis of U6 snRNA as a loading control for transcription shutoff/mRNA decay analysis of CRIP1, LSM8, and SPTSSA amino acid stretch reporters in Fig 2G. (C) (top) High resolution polyacrylamide northern blot analysis of SPTSSA amino acid stretch reporters. AF arrow indicates transcripts with full-length polyA tails; A0 arrow indicates fully deadenylated transcript. Timepoints represent time elapsed after shutoff of transcription with doxycycline. (bottom) Barplots showing distribution of northern blot signal over time ranging from full-length polyA tail (AF) to bulk polyA tail removal (A0) and separated into four bins of equal length. Lineplot of relative full-length polyA tail remaining over time is shown at the far right. (PDF) [file pone.0228730.s003.pdf]

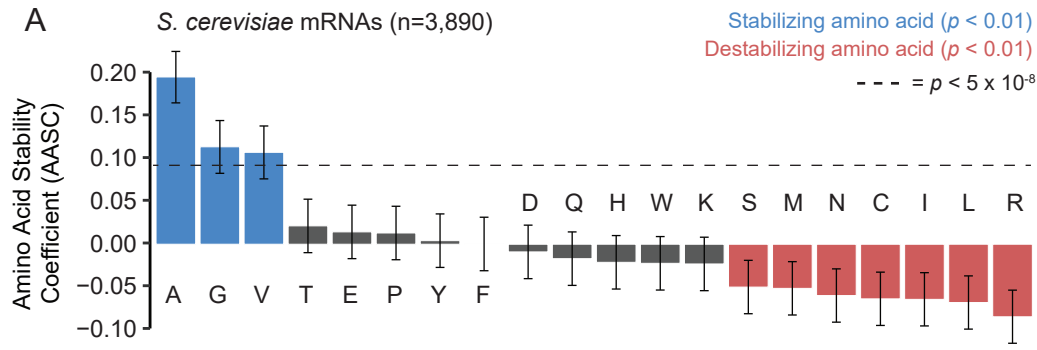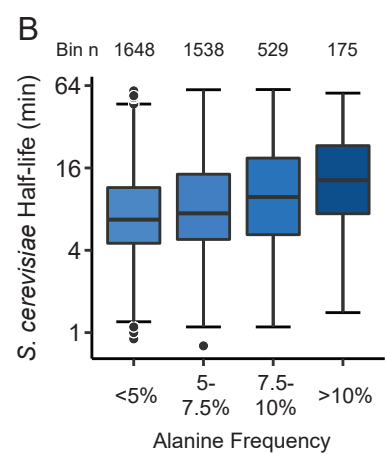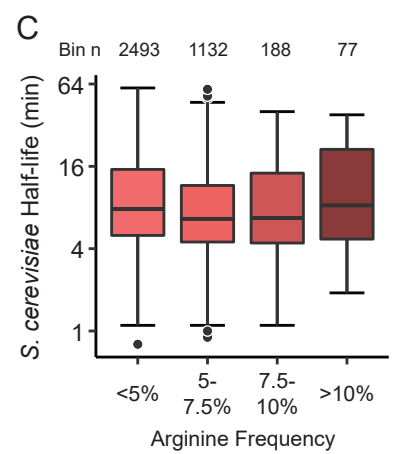

Supplement: S3 Fig — (A) Plot of amino acid stabilization coefficients (AASC) for S. cerevisiae mRNAs (n = 3,890). Blue represents significantly stabilizing amino acids; red represents significantly destabilizing amino acids (Pearson correlation test; p < 0.01). Dotted line indicates genome-wide significance level (p < 5 x 10^−8^; note that no destabilizing amino acids reached this significance level). Error bars indicate confidence interval about Pearson R estimate. (B) Boxplots of S. cerevisiae mRNA half-life distributions binned by alanine frequency. Number of transcripts in bin is indicated above each boxplot. Overall difference in means p < 2.2 x 10^−16^ (Kruskal-Wallis test). (C) Boxplots of S. cerevisiae mRNA half-life distributions binned by serine frequency. Number of transcripts in bin is indicated above each boxplot. Overall difference in means p = 8.5 x 10^−12^ (Kruskal-Wallis test). See also S2 Table for AASC values and statistical data and S3 Table for half-life data source. (PDF) [file pone.0228730.s004.pdf]

A

|       | Optimal | Non-Optimal | Total | Prob(Opt) | 95% CI       |
|-------|---------|-------------|-------|-----------|--------------|
| GC3   | 16      | 15          | 31    | 0.516     | 0.345-0.683  |
| AT3   | 14      | 16          | 30    | 0.467     | 0.299-0.642  |
| Total | 30      | 31          | 61    |           | $p = 0.7997$ |

B

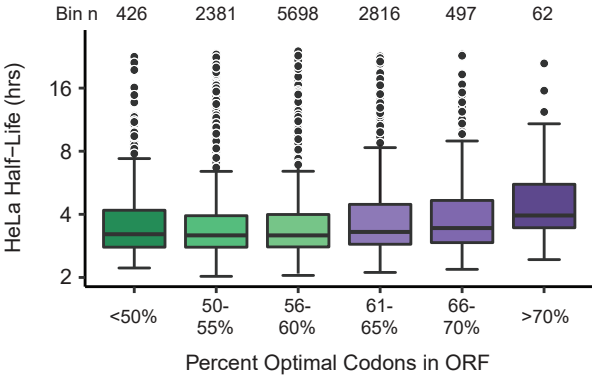

C

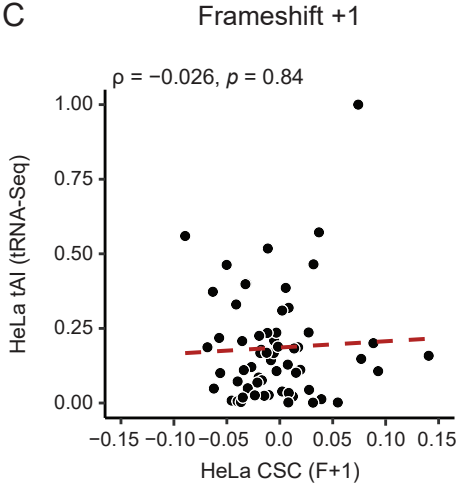

D

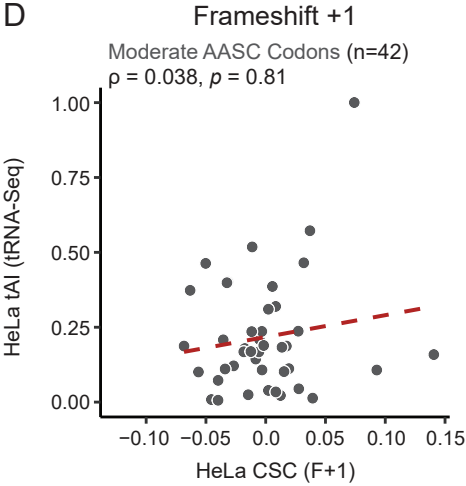

E

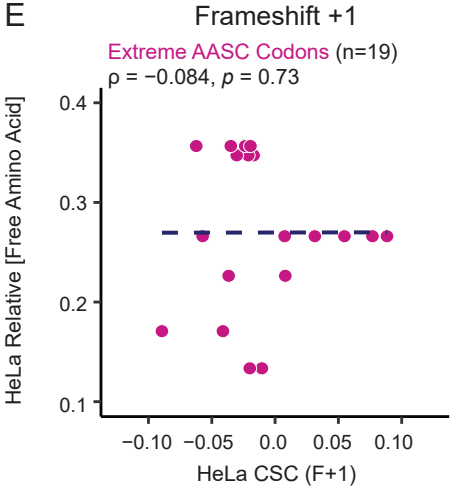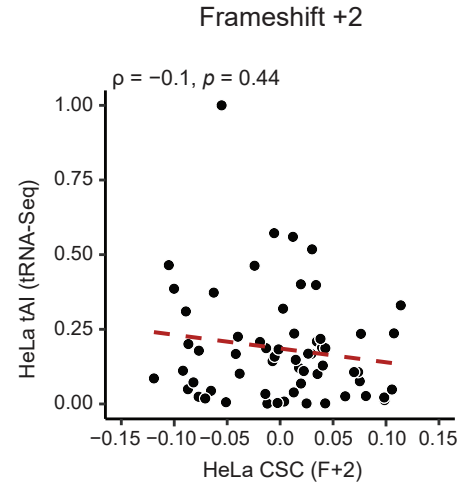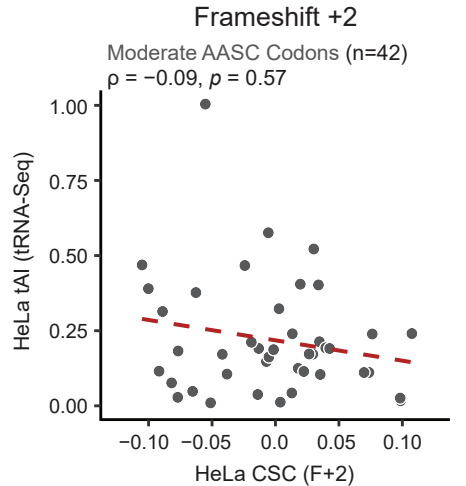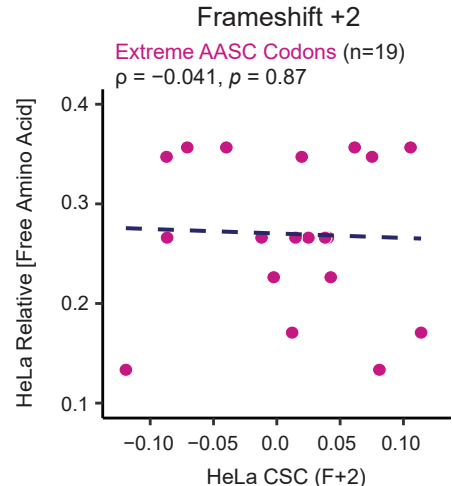

Supplement: S4 Fig — (A) Two by two table detailing proportions of optimal vs non-optimal codons (as defined by HeLa tRNA sequencing) with G or C at the at the 3’-nucleotide position (GC3) versus A or T (AT3). P-value and confidence intervals indicate results of Fisher’s exact test for difference in proportion of GC3 versus AT3 codons designated as optimal. (B) Boxplots of HeLa mRNA distributions binned by total optimal codon frequency (sum of codon frequencies for optimal codons, as defined in Fig 4A). Overall difference in means p < 2.2 x 10^−16^ (Kruskal-Wallis test). (C) Scatterplots of HeLa tAI versus CSCs calculated from ORF sequences frame-shifted by one nucleotide (F+1; top) and ORF sequences frame-shifted by two nucleotides (F+2; bottom) for all 61 codons. Spearman rho (ρ) and P-values indicate results of Spearman correlation test. (D) Scatterplots of HeLa tAI versus CSCs calculated from ORF sequences frame-shifted by one nucleotide (F+1; top) and ORF sequences frame-shifted by two nucleotides (F+2; bottom) for the 42 codons encoding amino acids with moderate effects on mRNA stability (-0.10 < AASC < 0.10, p > 10−30). Spearman rho (ρ) and P-values indicate results of Spearman correlation test. (E) Scatterplots of HeLa intracellular amino acid levels versus CSCs calculated from ORF sequences frame-shifted by one nucleotide (F+1; top) and ORF sequences frame-shifted by two nucleotides (F+2; bottom) for the 19 codons encoding the 6 amino acids with extreme effects on mRNA stability (AASC > 0.10 or < -0.10; p < 10−30). Spearman rho (ρ) and P-values indicate results of Spearman correlation test. (PDF) [file pone.0228730.s005.pdf]

A

HeLa mRNAs (n = 11,880)

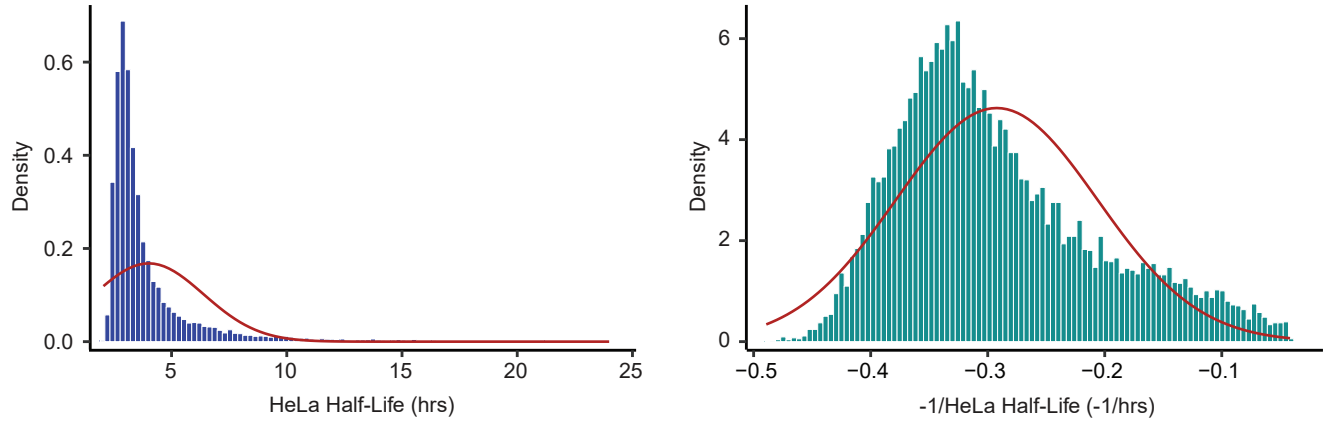

B

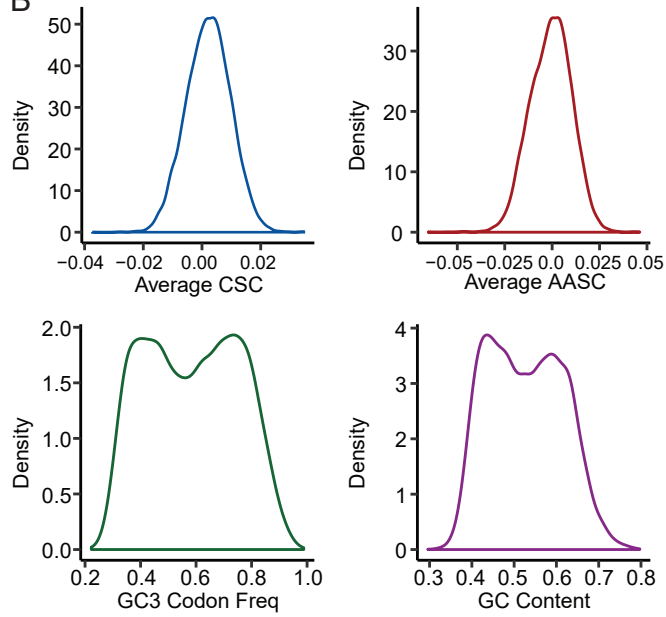

C

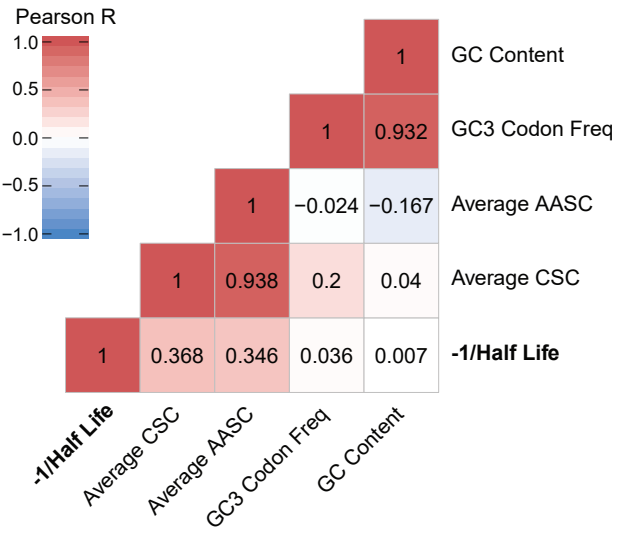

D

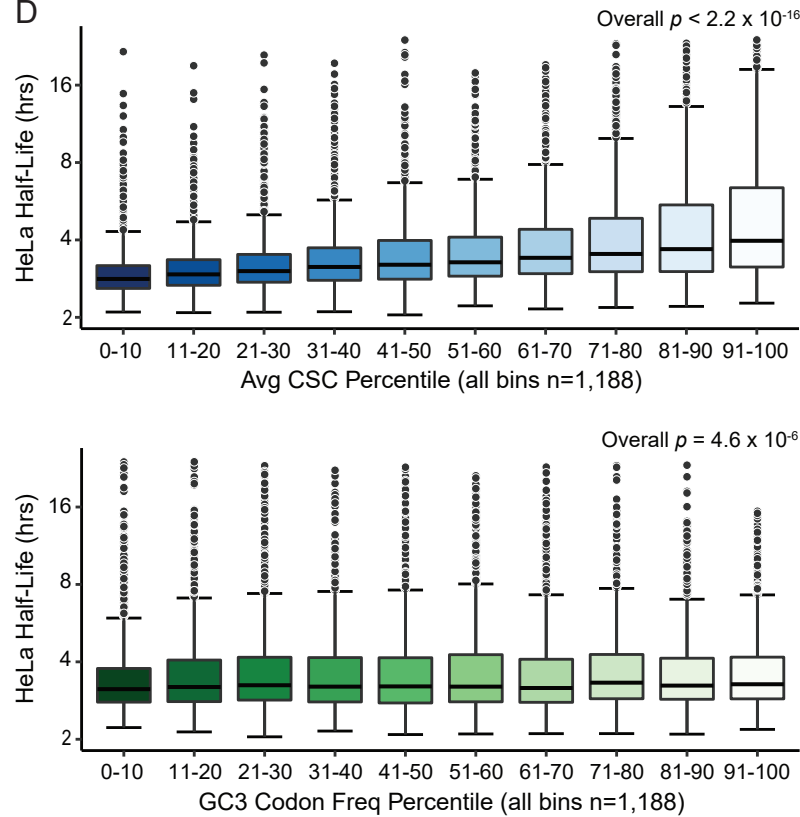

Supplement: S5 Fig — (A) Histograms showing distribution of untransformed HeLa endogenous mRNA half-lives (left) and HeLa half-lives after reciprocal transformation (-1/half-life; right). Red curve indicates a theoretical normal distribution with identical mean and standard deviation for each dataset. (B) Density plots showing distribution of values for transcript-average CSC, transcript-average AASC, GC3 codon frequency, and GC content. (C) Correlation matrix showing Pearson correlation estimates for each combination of the five indicated variables. -1/Half-Life = transformed HeLa mRNA half-lives. GC3 Codons = combined frequency of codons with G or C at the 3’-position. (D) Boxplots comparing HeLa half-life distributions for increasing transcript-average CSC (top) and GC3 codon frequency (bottom). P-values indicate overall difference in means across all bins (Kruskal-Wallis test). (PDF) [file pone.0228730.s006.pdf]

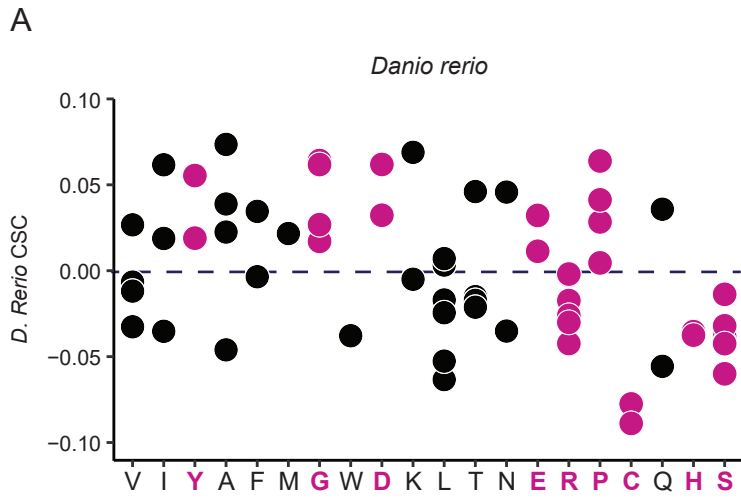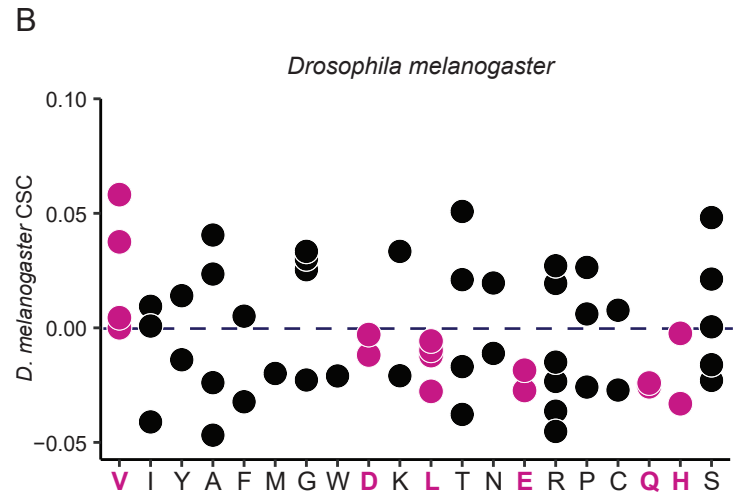

Supplement: S6 Fig — (A-B) Plots of codon stability coefficients (CSC) grouped by encoded amino acid for (A) Danio rerio (zebrafish) and (B) Drosophila melanogaster. Magenta indicates amino acids encoded exclusively by stabilizing (CSC > 0) or destabilizing (CSC < 0) synonymous codons. See also Fig 6C for phylogenetic tree diagram, S2 Table for species-specific CSC values, and S3 Table for half-life dataset sources. (PDF) [file pone.0228730.s007.pdf]
